# The low-complexity domains of the KMT2D protein regulate histone monomethylation transcription to facilitate pancreatic cancer progression

**DOI:** 10.1186/s11658-021-00292-7

**Published:** 2021-11-10

**Authors:** Weihan Li, Lei Wu, Hui Jia, Zenghua Lin, Renhao Zhong, Yukun Li, Chenwei Jiang, Shifan Liu, Xiaorong Zhou, Erhao Zhang

**Affiliations:** 1 Department of Immunology, School of Medicine, Nantong University, Nantong, 226001 People’s Republic of China; 2 Laboratory of Medical Science, School of Medicine, Nantong University, Nantong, 226001 People’s Republic of China; 3 Department of Hematology, Affiliated Hospital of Nantong University, Nantong University, Nantong, 226001 People’s Republic of China

**Keywords:** KMT2D, Low-complexity domain, Liquid–liquid phase separation, H3K4 monomethylation, Epigenetic therapy

## Abstract

**Background:**

Liquid–liquid phase separation (LLPS) within the nucleus is directly linked to driving gene expression through transcriptional complexes. Histone lysine methyltransferase 2D (KMT2D) is widely present in many cancers. It is known to epigenetically stimulate the expression of genes associated with tumorigenesis and metastasis. Our analyses show that KMT2D possesses two distinct low-complexity domains (LCDs) capable of driving the assembly of membrane-less condensates. The dependence of the mechanisms underlying monomethylation of H3K4 on the LLPS microenvironment derived from KMT2D LCDs is unclear in tumor.

**Methods:**

KMT2D LCD-depletion cells were used to investigate tumor cell proliferation, apoptosis, and migration. We identified some core proteins, including WDR5, RBBP5, and ASH2L, which are involved in the KMT2D-associated catalytic complex in KMT2D LCD-deficient cells to further elucidate the mechanism that decreases monomethylation of H3K4. We also evaluated the viability of KMT2D LCD-deficient cells in vivo. Finally, using 1,6-hexanediol (HD), an inhibitor of LLPS, we determined cell activities associated with KMT2D function in wild-type PANC-1 cells.

**Results:**

Without the LLPS microenvironment in KMT2D LCD-deficient cells or wild-type PANC-1 cells treated with HD, the WDR5 protein was significantly less stable and the protein–protein interactions between the components of the KMT2D–enzyme complex were attenuated, impairing the formation of the complex. Moreover, with the decrease in H3K4me1 level at enhancers, transcription factors such as LIFR and KLF4 were markedly downregulated, effectively inhibiting tumor progression. In xenograft tumor models, PANC-1 cells lacking the KMT2D LCDs showed effectively suppressed tumor growth compared to normal cells.

**Conclusions:**

Our data indicate that the two low-complexity domains of the KMT2D protein could form a stable LLPS microenvironment, promoting the KMT2D catalysis of H3K4 monomethylation through stabilization of the WDR5 protein and KMT2D–enzyme complex. Therefore, finding ways to regulate the LLPS microenvironment will be benefitial for new cancer treatment strategies.

**Supplementary Information:**

The online version contains supplementary material available at 10.1186/s11658-021-00292-7.

## Introduction

Methylation of histone 3 lysine 4 (H3K4) occurs widely in the mammalian genome. In this state, it is often associated with actively transcribed genes. For example, monomethylated H3K4 (H3K4me1) is found at gene enhancers and di- and tri-methylated H3K4 (H3K4me2 and H3K4me3, respectively) are located around active gene promoters [1, 2]. Post-translational histone modification yields considerable epigenetic regulatory elements in mammalian cells and plays a crucial role in controlling associated target gene expression [3]. Histone lysine methyltransferase 2D (KMT2D) is a critical histone H3K4 monomethyltransferase that can epigenetically stimulate gene expression in various signaling pathways associated with tumorigenesis and metastasis [4, 5]. Previous results indicate that the KMT2D protein promotes tumorigenesis via dysregulation of enhancer activity, disrupting normal developmental processes and altering gene networks regulated by tumor suppressors or oncoproteins [6, 7]. More recently, genes encoding KMT2D proteins have been implicated in many cancers. For instance, Lv et al. identified that KMT2D expression could promote prostate cancer proliferation and metastasis via the epigenetic transcriptional activation of leukemia inhibitory factor receptor (LIFR) and Kruppel-like factor-4 (KLF4), respectively via the PI3K/Akt and epithelial–mesenchymal transition-associated (EMT-associated) pathways [8]. Moreover, some research demonstrated that KMT2D is essential for tumor cell proliferation in many solid tumors, including pancreatic cancer, breast and colorectal cancer, and that KMT2D depletion increases the effectiveness of chemotherapy [9–12].

The human KMT2D protein is 5537 amino acids long, largely consisting of two clusters of plant homeotic domains (PHDs) in the N-terminus region, as well as an enzymatically active C-terminal SET domain [13]. Previous research revealed that the PHDs in the second cluster (PHD4–6) can recognize unmethylated H3 tails on the nucleosomes and that the C-terminal SET domain is critical for KMT2D-catalyzed H3K4 methylation activity and KMT2D protein stability [14–16]. The enzymatic function of KMT2D also depends on two phenylalanine and tyrosine-rich motifs (FYRN and FYRC) and an additional PHD domain located upstream of the SET domain. The protein also a high mobility group I (HMG-I) binding motif and multiple nuclear receptor-interacting motifs (LXXLLs), which are frequently found in transcription factor complexes [17–19]. However, whether other structures of KMT2D protein are vital for gene transcription in tumor cells remains unclear, and the mechanism remains to be comprehensively determined.

Liquid–liquid phase separation (LLPS) is mediated by multivalent proteins that harbor intrinsically disordered regions (IDRs) or stretches of low-complexity domains (LCDs) with repeated interaction motifs. It is closely related to a fundamental mechanism for driving the assembly of membrane-less condensates [20–24]. Interestingly, LLPS within the nucleus is directly linked to gene activation, potentially mediating gene expression through a transcriptional complex formed by mediator condensates assembled at the enhancer region of the gene clusters [25–29]. Similarly, recent studies indicated that the transcription complex or elongation complex in a gene’s promoter or enhancer regions depends on the LLPS microenvironment. For instance, Guo et al. suggested that phosphorylation of the Pol II C-terminal domain is involved in switching the Pol II location from an LLPS-induced condensate associated with transcription initiation to another condensate involved in RNA processing, implying that the LLPS microenvironment is essential for gene expression [30].

Generally, proteins composed of multiple LCD sequences could readily form an LLPS microenvironment during physiological processes. Although KMT2D is heavily associated with the methylation level of histones and the regulation of many transcription factors that affect tumor progression, the functions of many of its structures remain poorly studied. There is currently a poor understanding of the mechanisms underlying monomethylation of H3K4 at enhancers catalyzed by the KMT2D–enzyme complex, which depend on the LLPS microenvironment derived from the KMT2D LCDs.

Here, we show that the KMT2D protein contains two typical LCD structures that are key to the increased proliferation and migration of tumor cells. Our findings further demonstrate that the LCD domains could form a stable LLPS microenvironment that is crucial for the stability of WDR5 protein and the formation of the KMT2D–enzyme complex. Furthermore, our findings reveal the importance of the LLPS microenvironment for H3K4me1 formation. We also reveal the mechanism whereby LCDs regulate H3K4 monomethylation.

## Materials and methods

### Construction of plasmids

Two single-stranded sgRNA primers with complementary sequences were annealed in 5× annealing buffer consisting of 50 mM Tris-Cl (pH 7.5–8.0), 250 mM NaCl and 5 mM EDTA at 90 °C for 20 min. They were then cloned into BspQI restriction sites of the epiCRISPR plasmid, which was provided by Prof. Yihui Fan of Nantong University. The engineered sgRNA plasmids were tested and validated via sequencing for correctness (GENEWIZ). The sgRNA primers used in this study are listed in Additional file 1: Table S1.

To create the overexpression plasmids, the full length of the KMT2D gene was subcloned into the pcDNA3.1(-) vector using the XbaI and HindIII restriction sites (pcDNA3.1-KMT2D).

### Cell culture and transfection

We obtained cells of the cell lines HEK293T and PANC-1 (pancreatic cancer cells) from the American Type Culture Collection (ATCC). All cells were maintained in Dulbecco’s modified Eagle’s medium (DMEM; Hyclone) supplemented with 10% (v/v) fetal bovine serum (FBS; Biological Industries), 100 U/mL penicillin, and 100 µg/mL streptomycin (NCM Biotech). All the cell lines were incubated at 37 °C in a humidified atmosphere with 5% carbon dioxide.

Twenty-four hours prior to transfection, 5 × 10^5^ cells/well were cultured in 6-well plates and transfected with sgRNA plasmids using Lipofectamine 2000 (Invitrogen) according to the manufacturer’s protocol. Then, KMT2D-LCD1- and KMT2D-LCD2-deletion cells were transfected with KMT2D full-length plasmids to respectively yield OE1 and OE2 cells.

### Cell genotyping

Genomic DNA was harvested using a Mouse Director PCR Kit (Bimake). PCR amplification of KMT2D-LCDs was performed using 2 × Taq Master Mix (Dye Plus; Vazyme). PDLIM7 was used as the positive control to determine the concentration of high-quality DNA isolated from the modified cells. All primers in this study are listed in Additional file 1: Table S1.

### RT-PCR

Total RNA was isolated using Total RNA Extraction Reagent (Vazyme) and reverse-transcribed using HiScript II Q RT SuperMix for qPCR (Vazyme). Then, quantitative real-time PCR was performed using AceQ Universal SYBR qPCR Master Mix (Vazyme). All trials using reagents were performed according to the manufacturer’s instructions. Reactions were carried out in triplicate in 96-well optical reaction plates for 40 cycles, each at 95 °C for 10 s and 60 °C for 30 s. GAPDH was used as an internal control for normalization to determine the gene expression levels. All primers in this study are listed in Additional file 1: Table S1.

### Flow cytometry

All cells were harvested and washed three times with phosphate-buffered saline (PBS), then incubated with Annexin V-PE (Vazyme) for 15 min at 37 °C in the dark. Subsequently, all cells were washed with FACS wash buffer (1 × PBS containing 0.5% BSA and 0.03% sodium azide), and apoptotic cells were analyzed via flow cytometry (BD Calibur).

### Cell proliferation and scratch wound-healing assays

For the cell proliferation assay, 1 × 10^4^ transfected cells/well were seeded in 96-well plates. After incubation for 1, 3 or 5 days, 10 µL of CCK-8 (MultiSciences) diluted in 100 µL of RPMI-1640 basic medium (Hyclone) was added to each well to replace the conditioned culture medium. They were then incubated for another 2 h, and the plates were read at 450 nm.

For the scratch wound-healing assay, 5 × 10^5^ transfected cells/well were cultured in 6-well plates for 24 h, then a longitudinal scratch was made with a 10 µL pipette. They were incubated with 1% FBS-containing medium for another 48 h.

### Western blotting and immunoprecipitation assays

For western blotting analysis, the proteins were extracted from modified cells with a protein lysis buffer containing 1 M Tris-Cl (pH 7.4), 1% (v/v) NP-40, 3 M NaCl, 0.5 M EDTA, 1% (v/v) TritonX-100 and 0.25 % (m/v) sodium deoxycholate. Western blotting was performed using primary antibodies: KMT2D (Invitrogen, PA5-49581), Bcl2 (Santa Cruz), Bax (Santa Cruz), CDH1 (Service), EpCAM (Service), CDH2 (Service), vimentin (Service), LIFR (MultiSciences), KLF4 (Biovision), H3 (Abcam), H3K4me1 (Abcam), ASH2L (Santa Cruz), RBBP5 (Biovision), WDR5 (Biovision) and GAPDH (Santa Cruz).

For the immunoprecipitation assays, whole-cell lysates were prepared as described above. First, 40 µL of protein A-Agarose (Santa Cruz) was added for 2 h at 4 °C. The input sample was 150 µL of supernatant, and the remainder was used for the experimental groups. KMT2D antibody (Atlas Antibodies, HPA035977) or isotype control (normal rabbit IgG, Beyotine) was added, and the mixture was incubated at 4 °C overnight. Then, the pellet was harvested and washed three times with the protein lysis buffer and stored at −80 °C for further analysis.

### Cell immunofluorescence staining

All the transfected cells (1 × 10^5^ cells/well) were plated on sterile glass coverslips in 24-well plates. The next day, all the cells were fixed, permeabilized and blocked. Subsequently, the cells were stained overnight with primary fluorescent antibodies to KMT2D (Atlas Antibodies, HPA035977), H3K4me1, WDR5, EpCAM and CDH2. Then, the cells were incubated with secondary fluorescent antibodies, 488-labeled goat anti-rabbit IgG (H + L) (Beyotime), Cy3-labeled goat anti-mouse IgG (H + L) (Beyotime), and Hoechst (Beyotime, 5 µg/mL).

### Protein stability assays

After 24 h of culture in a 6-well plate, cycloheximide (CHX, Sigma-Aldrich) was added to each well at a concentration of 50 µg/mL. For the PANC-1 cells transfected with sgVector or sgRNA A1×B2 plasmids, the protein was isolated after 0, 2, 4, 8 or 10 h of CHX treatment. Simultaneously, PANC-1 cells transfected with sgVector or sgRNA A1×B2 plasmids were incubated with MG132 (MedChemExpress) at 10 µM for 10 h. The wild-type PANC-1 cells were treated with 1,6-hexanediol (10 mg/mL, Sigma-Aldrich) for about 2 h, CHX was added for 0, 2 or 4 h, then the protein was harvested and the expression of WDR5 was determined using western blotting assays.

### 1,6-hexanediol treatment trials

For western blotting and real-time PCR assays, wild type PANC-1 cells (5 × 10^5^ cells/well) were cultured in 6-well plates, and treated with increasing concentrations of 1,6-hexanediol (0, 2, 4, 10, 15 or 20 mg/mL) or for increasing durations (0, 2, 4, 6, 8 and 10 h) as indicated in the relevant figures. For immunofluorescence staining and immunoprecipitation assays, the wild-type PANC-1 cells were treated with CHX for 6 h at a concentration of 10 mg/mL.

### Animal experiments

All the animal experiments were approved by the Animal Care and Use Committee of Nantong University. Female Balb/c-nude mice (4–6 weeks old, 18–20 g) were purchased from the Laboratory Animal Center of Nantong University. PANC-1 cells transfected with sgVector (n = 5) or sgRNA A1×B2 (n = 5) were injected subcutaneously (6 × 10^6^ cells per mouse) into the left flanks of the mice. After 4 weeks, we carefully observed the tumor formation, verifying the activity of the cells in vivo.

### Statistical analysis

Statistical significance was calculated using GraphPad software. For this paper, CCK8 and real-time PCR data were analyzed using the two-tailed Student’s t test. In general, n.s. indicates not significant, and *, ** and *** respectively indicate p values less than 0.05, 0.01 and 0.001. In Figs. 2, 3, 4 and 5, the statistical significance was calculated based on the “KMT2D-LCDs knockout group” vs. “vehicle group”, or “HD treatment group (HD+)” vs. “no treatment group (HD-)”.

## Results

### KMT2D structural analysis and functional role in tumor progression

**Fig. 1:**
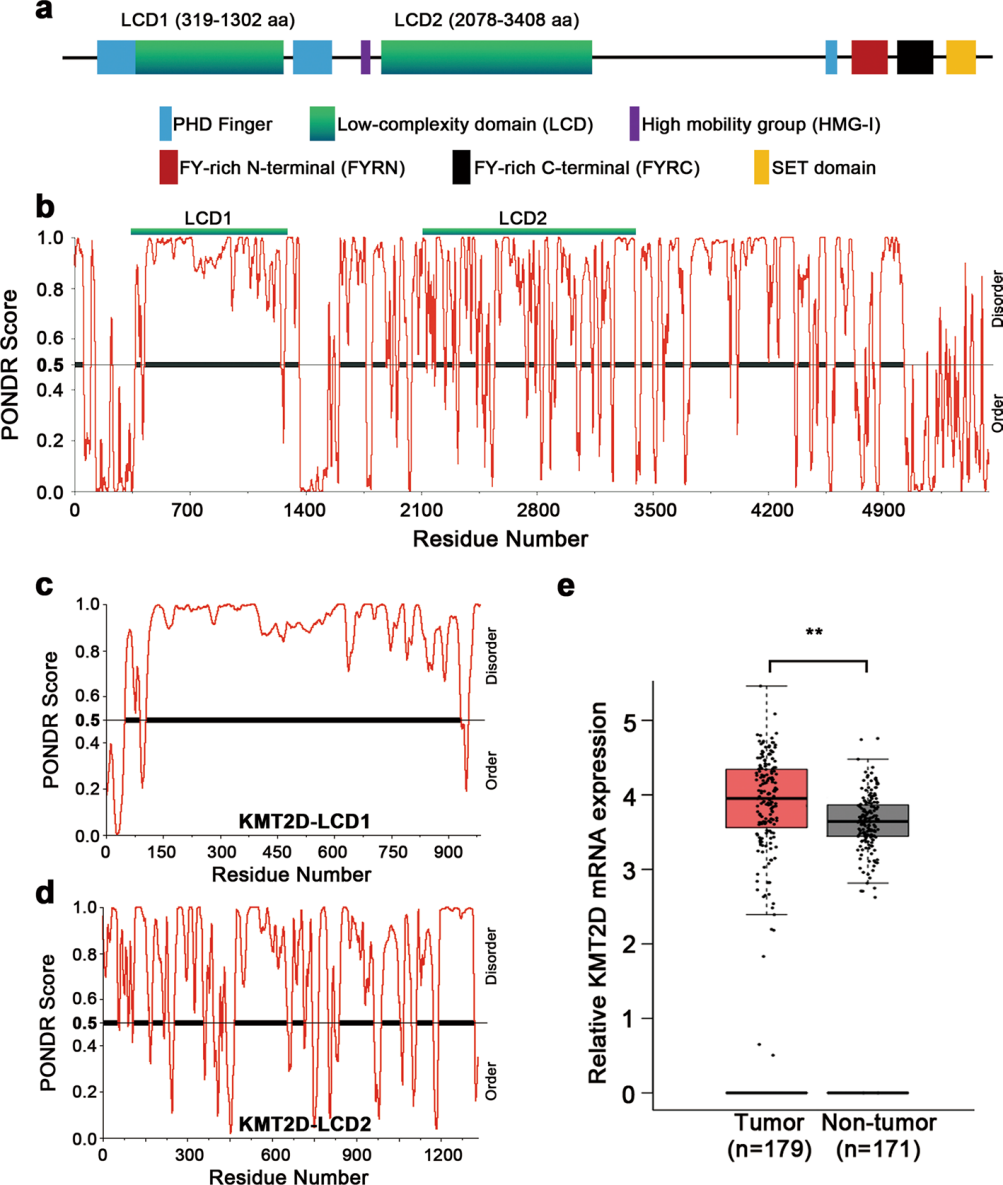
Structural and functional insights into the KMT2D protein. **a** Schematic representation of the domain structure for KMT2D protein. KMT2D contains two clusters of plant homeotic domains (PHDs) in the amino-terminal region. The second cluster can recognize unmethylated histone H4 tails. On the C terminus, FY-rich N-terminal (FYRN) and FYR C-terminal (FYRC) and an enzymatically active SET domain are closely connected to generate a functional and stable protein. A high mobility group I (HMG-I) binding motif (frequently found in transcription factors and cofactors) and a PHD domain are present in the protein sequences. The two domains of interest are indicated as LCD1 and LCD2. **b**–**d** Analyses of KMT2D, KMT2D LCD1, and KMT2D LCD2 using the PONDR database. KMT2D protein has two intrinsically disordered regions with multi-phosphorylation sites and a high proportion of proline and glutamine. **e** Analyses of KMT2D expression in pancreatic cancer (Tumor, n = 179) and normal tissue (Not-tumor, n = 171) based on previously published gene expression array data listed in The Cancer Genome Atlas (TCGA) database. In contrast to normal tissues, significant overexpression of KMT2D was found in pancreatic cancer tissues (p < 0.01)

KMT2D is in the trithorax-related (Trr-related) subgroup of the histone–lysine N-methytransferase 2 (KMT2) family. The wild-type protein could be required for cancer cell survival in many cancers. For our investigation of cancer survival-related mechanisms, we first analyzed the protein structure of KMT2D, which consists of many known components, such as plant homeotic domains (PHDs), high mobility group I (HMG I), FY-rich N-terminal (FYRN), FY-rich C-terminal (FYRC), and SET domain (Fig. 1a).

The Predictor of Natural Disordered Regions (PONDR, http://www.pondr.com/) analysis revealed that the KMT2D protein has two distinct structures with low random complexity at 319 aa to 1302 aa and 2078 aa to 3408 aa, respectively named LCD1 and LCD2 (Fig. 1b). Our analyses show that LCD1 has 150 phosphorylation sites, namely 128 phosphoserine, 19 phosphothreonine and 3 phosphotyrosine sites, and 15 repeats of a 5-amino acid structure (S/P-P-P-E/P-E/A). Its ratio of proline and glutamine accounts for approximately 28.86% of its amino acids. This shows that LCD1 of the KMT2D protein could generate an LLPS microenvironment (Fig. 1c). Similarly, the LCD2 structure has 164 phosphorylation sites, namely 112 phosphoserine, 40 phosphothreonine and 12 phosphotyrosine sites, and 24.42% of its amino acids are proline and glutamine (Fig. 1d). These results indicate that the KMT2D protein could form a stable LLPS microenvironment based on the characteristics its LCD1 and LCD2 structures.

To determine the role of KMT2D in pancreatic carcinogenesis, we investigated the differential KMT2D expression based on previously published gene expression array data listed in the Cancer Genome Atlas (TCGA) and Gene Expression Omnibus (GEO) databases (Fig. 1e and Additional file 1: Fig. S1). Based on the analysis, we surmise that KMT2D protein might exert tumor-promoting properties in pancreatic cancer. However, the role of KMT2D LCDs in tumor survival needs further exploration through understanding the impact of the LLPS microenvironment on tumor progression.

### KMT2D LCDs knockout strategy and verification

**Fig. 2:**
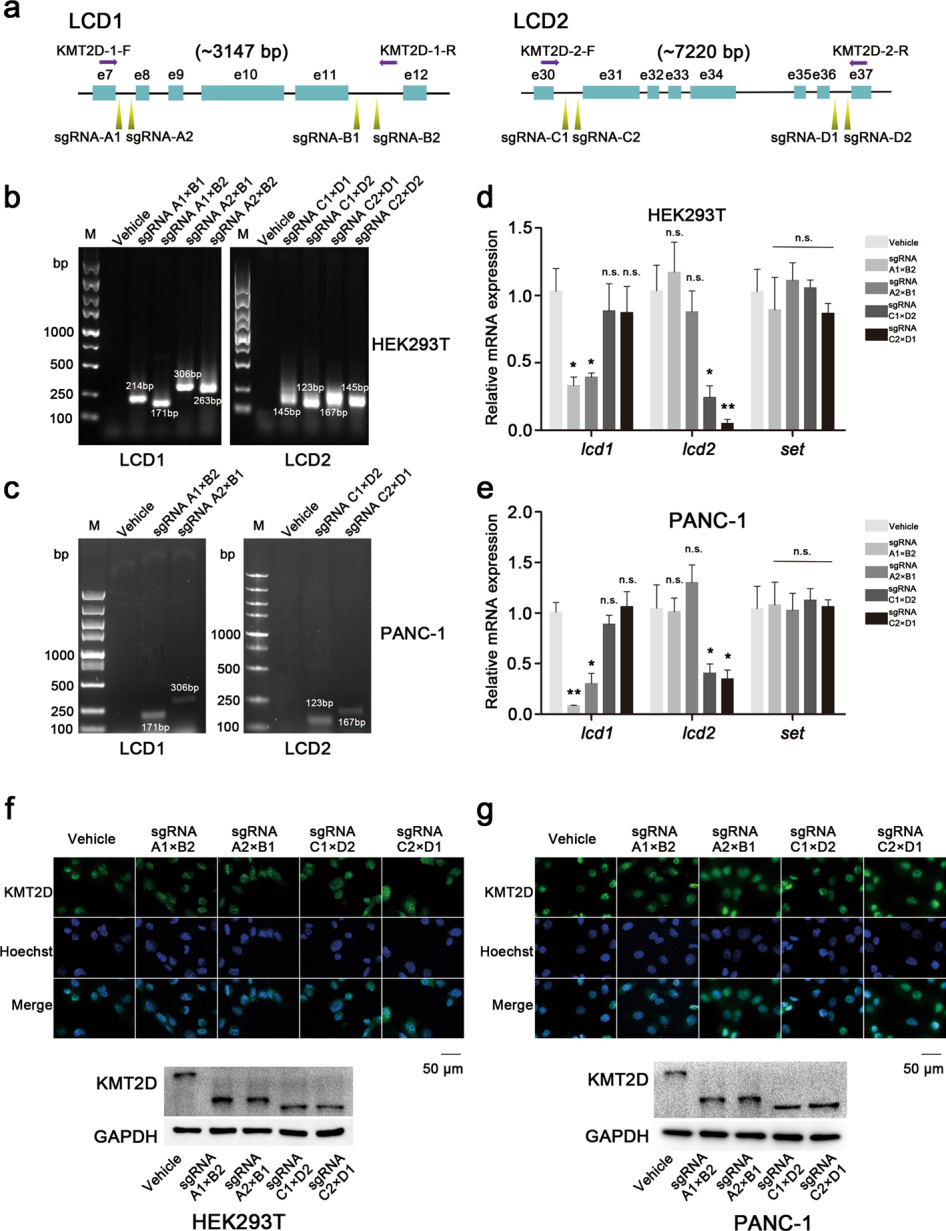
LCD knockout strategy and verification in HEK293T and PANC-1 cells. **a** Schematic representation of LCD knockout strategy using CRSPR-Cas9 technology. LCD1 (located between exon 8 and 11) was deleted with sgRNA primers: two upstream primers (sgRNA-A1/A2) and two downstream primers (sgRNA-B1/B2). This was then verified by genotyping with KMT2D-1-F/R primers. For LCD2 (located between exon 31 and 36), two upstream primers (sgRNA-C1/C2) and two downstream primers (sgRNA-D1/D2) were used to delete LCD2 and then the rest was tested with KMT2D-2-F/R primers. **b**, **c** Verification with genotyping assays. In HEK293T and PANC-1 cells, genotyping PCR was performed in transfected cells. The results are consistent with the predictions compared with the control group (vehicle). **d**, **e** RT-PCR analyses to measure LCD knockout. *lcd1* mRNA expression only significantly decreased in LCD1-deleted cells, while that of *lcd2* only in LCD2-deleted cells. The *set* gene located at the C-terminal of the KMT2D gene was selected as a control (error bars denote standard deviation). **f**, **g** Representative images of immunofluorescences and western blotting in HEK293T and PANC-1 cells. After LCD knockout, the KMT2D protein level did not significantly decrease, and KMT2D LCD1 or KMT2D LCD2 was successfully deleted. KMT2D, 593 KDa; LCD1 deletion-KMT2D, 483 KDa; LCD2 deletion-KMT2D, 447 KDa. All the values are represented as means ± SD. Data are representative of three independent experiments

To investigate LCD functional impact for KMT2D protein, the LCD1 and LCD2 structures were deleted using CRISPR-Cas9 technology. For LCD1 knockout, two upstream primers (sgRNA-A1 and sgRNA-A2) and two downstream primers (sgRNA-B1 and sgRNA-B2) were designed, and four combination knockouts, A1B1, A1B2, A2B1, and A2B2, were performed in HEK293T and PANC-1 cells (Fig. 2a). Similarly, for LCD2 knockout, two upstream primers (sgRNA-C1 and sgRNA-C2) and two downstream primers (sgRNA-D1 and sgRNA-D2) were designed, combined, and transfected in vitro (Fig. 2a). Then, two pairs of genotyping primers, KMT2D-1-F/R and KMT2D-2-F/R, were respectively used to validate the successful LCD1 knockout and LCD2 knockout in vitro.

The results show that combining sgRNA primers could effectively delete the LCD domain from KMT2D in HEK293T cells genetically, and similar results were obtained in the PANC-1 cell lines (Fig. 2b, c and Additional file 1: Fig. S2). Subsequently, we further validated the accurate knockout of LCDs in engineered cells via sequencing (data not shown). Next, we used RT-PCR analysis to measure the LCD mRNA expressions in various recombinant cells, and we selected the *set* gene, located at the C-terminal of the KMT2D gene, as a control for gene expression. The results show that in contrast to the control group (vehicle), *lcd1* mRNA expression was significantly lower than in HEK293T and PANC-1 cells modified with sgRNA A1B2 or sgRNA A2B1, while the mRNA expression of *lcd2* showed no significant change. Similarly, in sgRNA C1D2- or sgRNA C2D1-transfected cells, only *lcd2* mRNA expression significantly decreased (Fig. 2d, e). Moreover, the immunofluorescence results showed that KMT2D protein expression was not significant reduced in LCD knockout-modified HEK293T and PANC-1 cells (Fig. 2f, g). Interestingly, our results also indicate that the KMT2D protein in the cells modified with LCD knockout was highly scattered, while that of the control group (vehicle) was clustered. However, the western blotting assay results demonstrate that full-length or truncated KMT2D protein respectively expresses in wild-type or LCD knockout-modified cells. Our results indicate that the molecular weight of the full-length KMT2D protein is approximately 593 KDa, whereas the LCD1 deletion- and LCD2 deletion-KMT2D are respectively approximately 483 KDa and 447 KDa.

These results indicate that we successfully generated stable KMT2D LCD1- or LCD2-deletion cell lines in HEK293T and PANC-1 cells, providing the foundation for our subsequent experiments.

### LCD depletion inhibits cell proliferation and attenuates tumor migration ability

**Fig. 3:**
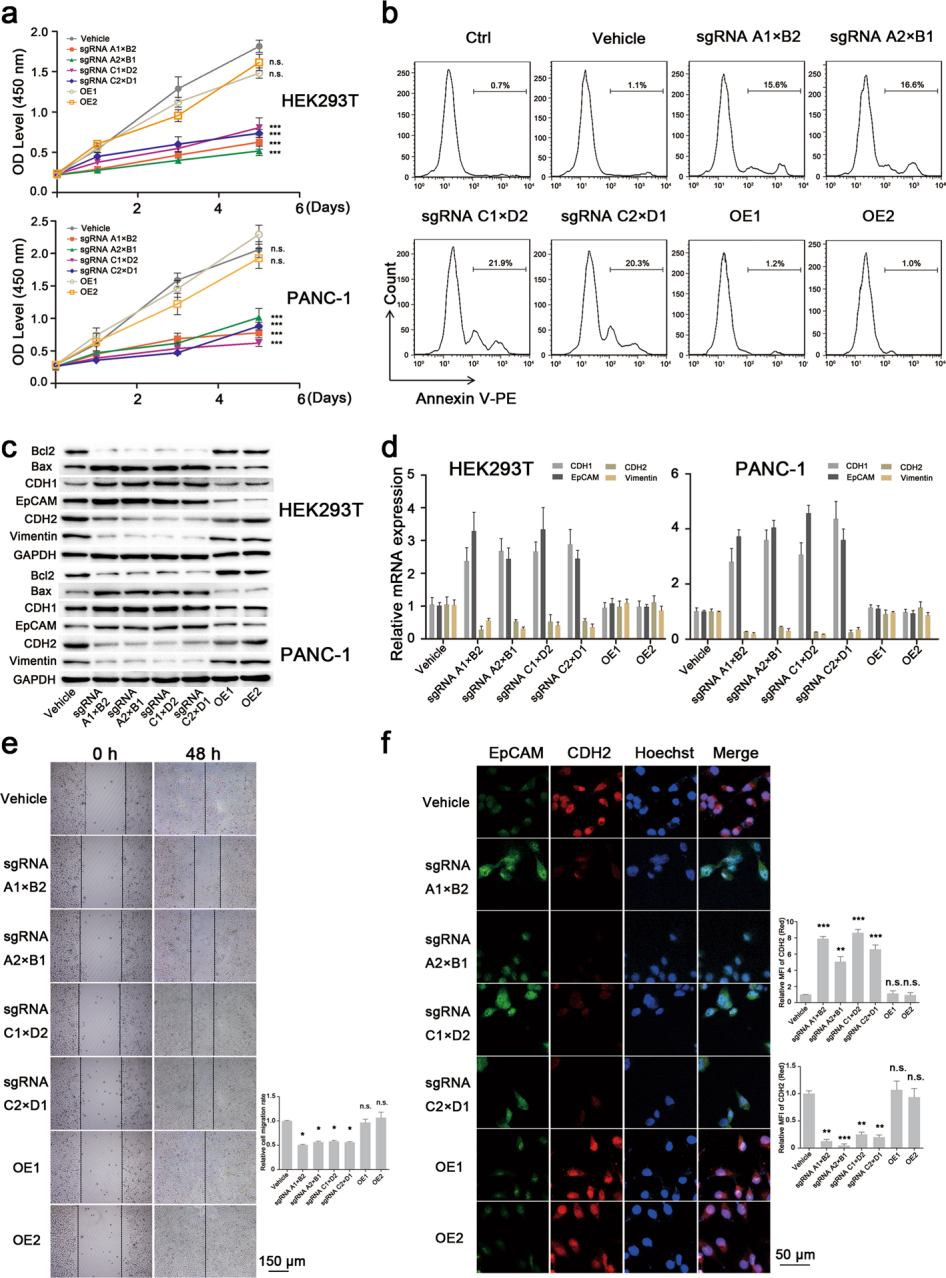
Effect of KMT2D LCD knockout on cell activities in HEK293T and PANC-1 cells. **a** Cell viability analysis of HEK293T and PANC-1 cells carrying vectors for the vehicle or KMT2D LCD knockout at 0, 24, 72 and 120 h post-transfection. Compared with the control group (vehicle), KMT2D LCD depletion significantly suppressed cell proliferation, while the OE1 cells (LCD1-depleted KMT2D cells re-expressing full-length KMT2D) and OE2 cells (LCD2-depleted KMT2D cells re-expressing full-length KMT2D) showed restored cell proliferation compared to the wild-type cells (error bars denote standard deviation). **b** Representative flow cytometry of cell apoptosis assays in PANC-1 cells. The apoptotic cell ratios were significantly increased in PANC-1 cells with KMT2D LCD deficiency. **c** Western blotting analyses of the apoptosis- and metastasis-related proteins after KMT2D LCD knockdown in HEK293T and PANC-1 cells. KMT2D LCD deficiency increased the Bax/Bcl2 ratio and the epithelial marker-to-mesenchymal marker ratio. Bcl2, 26 KDa; Bax, 20 KDa; EpCAM, 40 KDa; Vimentin, 57 KDa; GAPDH, 37 KDa. **d** RT-PCR assays of metastasis-associated markers. The gene expressions of epithelial markers (CDH1 and EpCAM) had markedly increased, while those of mesenchymal markers (CDH2 and vimentin) had decreased after KMT2D LCD depletion (error bars denote standard deviation). **e** Scratch wound-healing assays in PANC-1 cells. Knockout of KMT2D LCD significantly attenuated the cell migration ability. **f** Representative images of cell immunofluorescence determining the CDH2 and EpCAM expression in PANC-1 cells. After LCD knockout, CDH2 expression significantly reduced while that of EpCAM increased. All values are means ± SD. Data are representative of three independent experiments

We constructed OE1 and OE2 cells that respectively overexpress full-length KMT2D or KMT2D without the two LCDs (for verification, see Additional file 1: Fig. S3). Then, cell proliferation, apoptosis and migration were evaluated. As expected, KMT2D LCD depletion significantly suppressed cell proliferation compared to the control group (Fig. 3a). In KMT2D LCD-depleted HEK293T cells, the cell proliferation rate decreased by approximately 38.45% after 5 days of culture. A significant reduction of around 32.56% was also observed in KMT2D LCD-depleted PANC-1 cells (Fig. 3a). Moreover, in PANC-1 cells with KMT2D LCD depletion, the apoptotic cell ratios were significantly higher than for the control group (Fig. 3b). More importantly, the cellular activities were restored by re-expression of full-length KMT2D in KMT2D LCD-depleted cells, indicating the importance of the LCD domain in regulating such cellular activities.

We further observed that the expression of the anti-apoptotic proteins Bcl2 dramatically decreased in stable KMT2D LCD-depleted HEK293T and PANC-1 cells, while the Bax pro-apoptotic protein was more highly expressed than in the control (Fig. 3c). Overall, the resulting increase in the Bax/Bcl2 ratio in KMT2D LCD-deficient cells was shown to promote cell apoptosis.

Next, to further verify the role of KMT2D LCDs in cell metastasis, epithelial-to-mesenchymal transition (EMT) markers were detected in HEK293T and PANC-1 cells transfected with sgLCDs. Western blotting analyses showed that the expressions of the epithelial markers CDH1 (E-cadherin) and EpCAM were significantly higher in LCD-depleted cells while those of the mesenchymal markers CDH2 (N-cadherin) and vimentin were lower (Fig. 3c). Similarly, we observed that the gene expression of epithelial markers markedly increased after KMT2D LCD depletion, but the mRNA levels of the mesenchymal markers in KMT2D LCD-depleted cells had significantly decreased compared to the control group (Fig. 3d).

To further determine the role of KMT2D LCDs, scratch wound-healing assays were performed with KMT2D LCD-depleted cells. We found that the migratory ratio significantly decreased after KMT2D LCD knockout compared to the control (Fig. 3e), which further demonstrates cellular function impairment in KMT2D LCD-depleted cells. The expression change in EMT-associated markers measured using immunofluorescence assays showed a consistent trend with those of our previous western blotting and RT-PCR analyses (Fig. 3f). More importantly, the cell activities in OE1 and OE2 cells showed comparable results to those of PANC1 cells transfected with vehicle plasmids. Our results reveal that LCDs in the KMT2D protein play an essential role in tumor survival by promoting tumor cell proliferation and inhibiting tumor cell apoptosis.

### KMT2D modulates cell survival by stimulating LIFR and KLF4

KMT2D, a histone methyltransferase, can effectively catalyze monomethylation of H3K4 (to yield H3K4me1). Therefore, the abundance of H3K4me1 can be used as a marker to reflect the magnitude of KMT2D activity.

**Fig. 4:**
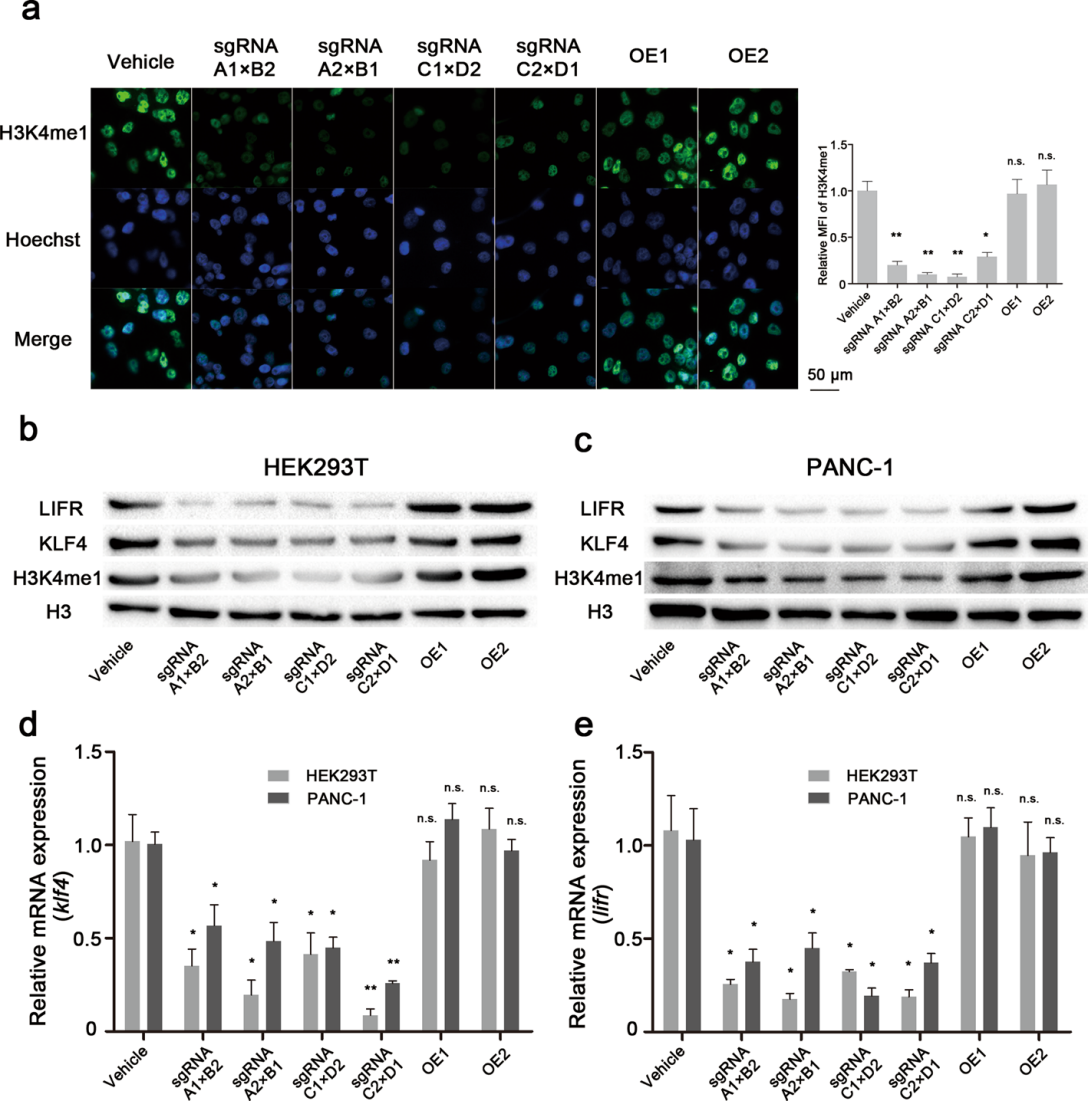
KMT2D increases LIFR and KLF4 expression in an LCD-dependent manner. **a** Representative images of cell immunofluorescence determining the H3K4me1 expression in PANC-1 cells. After LCD knockout, the H3K4me1 level was markedly reduced in the nucleus, but transfection of full-length KMT2D plasmids could reverse the decrease in H3K4me1 expression. **b**, **c** Western blotting analyses of the H3K4me1, LIFR, and KLF4 proteins in HEK293T and PANC-1 cells. Knockout of KMT2D LCD significantly attenuated the H3K4me1 expression and decreased LIFR and KLF4 protein expression. LIPR, 190 KDa; KLF4, 62 KDa; H3K4me1, 17 KDa; H3 (histone H3), 17 KDa. **d**, **e** RT-PCR analyses of LIFR and KLF4 expression. After LCD deletion in HEK293T and PANC-1 cells, the gene expression of LIFR and KLF4 decreased compared to the control group (vehicle; error bars denote standard deviation). All values are represented as means ± SD. Data are representative of three independent experiments

To investigate the effect of LCD depletion on the catalytic function of KMT2D, we used an immunofluorescence assay to quantify H3K4me1 abundance, finding that in the stable KMT2D LCD-depleted HEK293T and PANC-1 cell lines, the H3K4me1 level was remarkably reduced in the nucleus (Fig. 4a and Additional file 1: Fig. S4). Moreover, the protein expression of H3K4me1 was also evaluated using western blotting assays, and as expected, the protein level of H3K4me1 was notably lower than that in the control cells (Fig. 4b, c). Interestingly, the H3K4me1 expression in the OE1 and OE2 cells could be restored to the original level (same as the wild-type). Our findings reveal that KMT2D LCDs play an integral part in KMT2D catalytic activity and that LCD deficiency can significantly impair its methylation ability.

LIFR and KLF4 are respectively essential transcription factors for the PI3K/Akt signaling pathway and metastasis signaling pathway [31, 32]. Previously, Lv et al. demonstrated that KLF4 and LIFR were the associated target genes of KMT2D protein through H3K4me1 modification [8]. Herein, we found that the gene expressions of LIFR and KLF4 consistently decreased after KMT2D LCD depletion in HEK293T and PANC-1 cells, positively correlating with the H3K4me1 level (Fig. 4d, e). These results suggest that KMT2D LCDs could induce alterations in the proliferation and metastatic pathways by epigenetically activating LIFR and KLF4 in an H3K4me1-dependent manner.

### The LCDs of KMT2D control the stability of WDR5 protein and influence the formation of the KMT2D catalytic complex

**Fig. 5:**
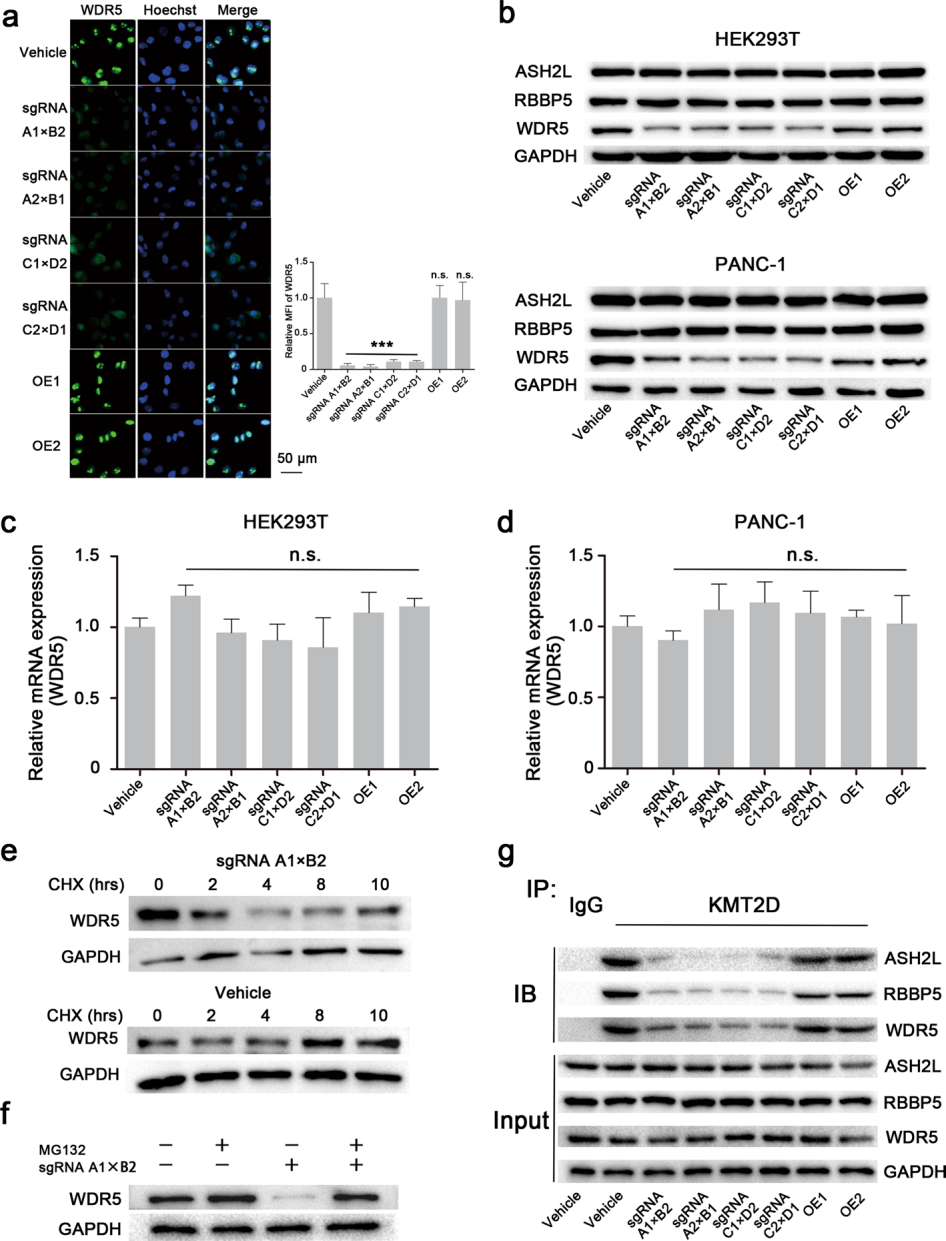
KMT2D LCDs modulate the stability of WDR5 protein and influence the formation of the KMT2D-enzyme complex. **a** Representative images of cell immunofluorescence detecting the WDR5 expression in modified-PANC-1 cells. The WDR5 level had markedly reduced in the LCD-depleted cells, but normal expression was recovered in OE1 and OE2 cells. **b** Western blotting analyses of the KMT2D-enzyme complex, including WDR5, ASH2L, and RBBP5, in HEK293T and PANC-1 cells. Knockout of KMT2D LCDs significantly attenuated WDR5 expression, but the expressions of ASH2L and RBBP5 were not affected. In OE1 and OE2 cells, WDR5 expression is restored. ASH2L, 69 KDa; RBBP5, 59 KDa; WDR5, 36 KDa; GAPDH, 37 KDa. **c**, **d** RT-PCR analyses of WDR5 gene expression. After LCD deletion in HEK293T and PANC-1 cells, the gene expression of WDR5 was similar to that in OE1 cells, OE2 cells, and control group (vehicle; error bars denote standard deviation). **e**, **f** Western blotting analyses of WDR5 protein expression with CHX or MG132 treatment. The WDR5 protein level in KMT2D LCD knockout cells gradually decreased with the time after CHX treatment compared with the wild-type cells, indicating that the stability of WDR5 protein is closely related KMT2D LCDs. **g** Co-immunoprecipitation analyses of the interactions between KMT2D and other components. The interactions between KMT2D and WDR5 were significantly reduced in KMT2D LCD-depleted PANC-1 cells. Similar results were found for the interactions of KMT2D with ASH2L and RBBP5. In OE1 and OE2 cells, these interactions were restored. All values are represented as means ± SD. Data are representative of three independent experiments

To further determine the mechanism underlying the decrease in expression of H3K4me1 in KMT2D LCD-deficient cells, the expressions of some core proteins involved in the KMT2D-associated catalytic complex were determined, including WD-repeat protein 5 (WDR5), retinoblastoma-binding protein 5 (RBBP5), and absent small homeotic 2 like (ASH2L). KMT2D is a major mammalian H3K4 monomethyltransferase with activity dependent on a stable protein complex associated with WDR5, RBBP5, ASH2L, dumpy-30 (DPY30), and nuclear receptor coactivator 6 (NCOA6) [18]. Immunofluorescence data shows that, in stable KMT2D LCD-depleted cell lines, the expression of WDR5 protein markedly decreased compared with KMT2D-integrated HEK293T and PANC-1 cells (Fig. 5a and Additional file 1: Fig. S5). Moreover, western blotting results reveal that WDR5 expression significantly decreased, while the expression of other core proteins of the stable protein complex, ASH2L and RBBP5, were unchanged in stable KMT2D LCD-depleted HEK293T and PANC-1 cell lines (Fig. 5b). Importantly, we found that the WDR5 protein could be restored to its original level in the OE1 and OE2 cells (same as wild type). Therefore, we speculate that the LCD domains of KMT2D play a vital role in maintaining WDR5 protein expression, giving stability to the KMT2D complex and promoting significant H3K4 monomethyltransferase activity.

Interestingly, we further showed that the transcription level of the WDR5 gene in stable KMT2D LCD-depleted HEK293T and PANC-1 cell lines are comparable to the control (vehicle). Although our results suggest that the KMT2D LCDs may affect the stability of WDR5 protein, no changes in the WDR5 mRNA levels were detected (Fig. 5c, d). Therefore, to further validate whether the LCDs of KMT2D affect the stability of WDR5 protein, a cycloheximide (CHX) chase assay was performed by adding CHX to KMT2D-integrated (vehicle) or KMT2D LCD-depleted PANC-1 cells. In the KMT2D LCD-depleted cells, we found that the half-life of the WDR5 protein level gradually decreased with time, while the wild-type cells were not affected by CHX treatment, indicating that the stability of WDR5 protein was dependent on the LCD domains of KMT2D (Fig. 5e). Furthermore, treating cells with the proteasome inhibitor MG132 led to accumulation of WDR5 protein in KMT2D LCD knockout cells, further substantiating our findings (Fig. 5f).

Next, we investigated whether the LCD domains influence the interactions between KMT2D and other components in the protein complex. Co-immunoprecipitation results showed that the interactions between KMT2D and WDR5 were significantly reduced in stable KMT2D LCD knockout PANC-1 cell lines while that interaction could be restored in the OE1 and OE2 cell lines. Similar results were also obtained when we compared the interactions of KMT2D with ASH2L and RBBP5 (Fig. 5g). Previous studies showed that WDR5 protein acts as a bridge to connect KMT2D and other components, supporting KMT2D catalytic complex formation [18]. Theoretically, with the weakening of the interaction between KMT2D and WDR5, the combination of KMT2D and other components would inevitably decrease, which is consistent with our findings. In summary, the LCD domains of KMT2D can significantly affect the stability of WDR5 protein and the interactions between KMT2D and other proteins in this catalytic complex, which can ultimately affect its specific methyltransferase activity.

### The LLPS microenvironment formed by KMT2D may influence its functions and modulate the properties of WDR5

According to our analyses, the LCDs of KMT2D possess a solid predisposition to LLPS formation. Therefore, we surmised that the LLPS microenvironment formed by KMT2D LCDs could significantly affect the enzymatic activity of the KMT2D protein. To that end, we treated relevant cell lines with 1,6-hexanediol (HD), an inhibitor of LLPS formation, and assessed their cellular activities.

**Fig. 6:**
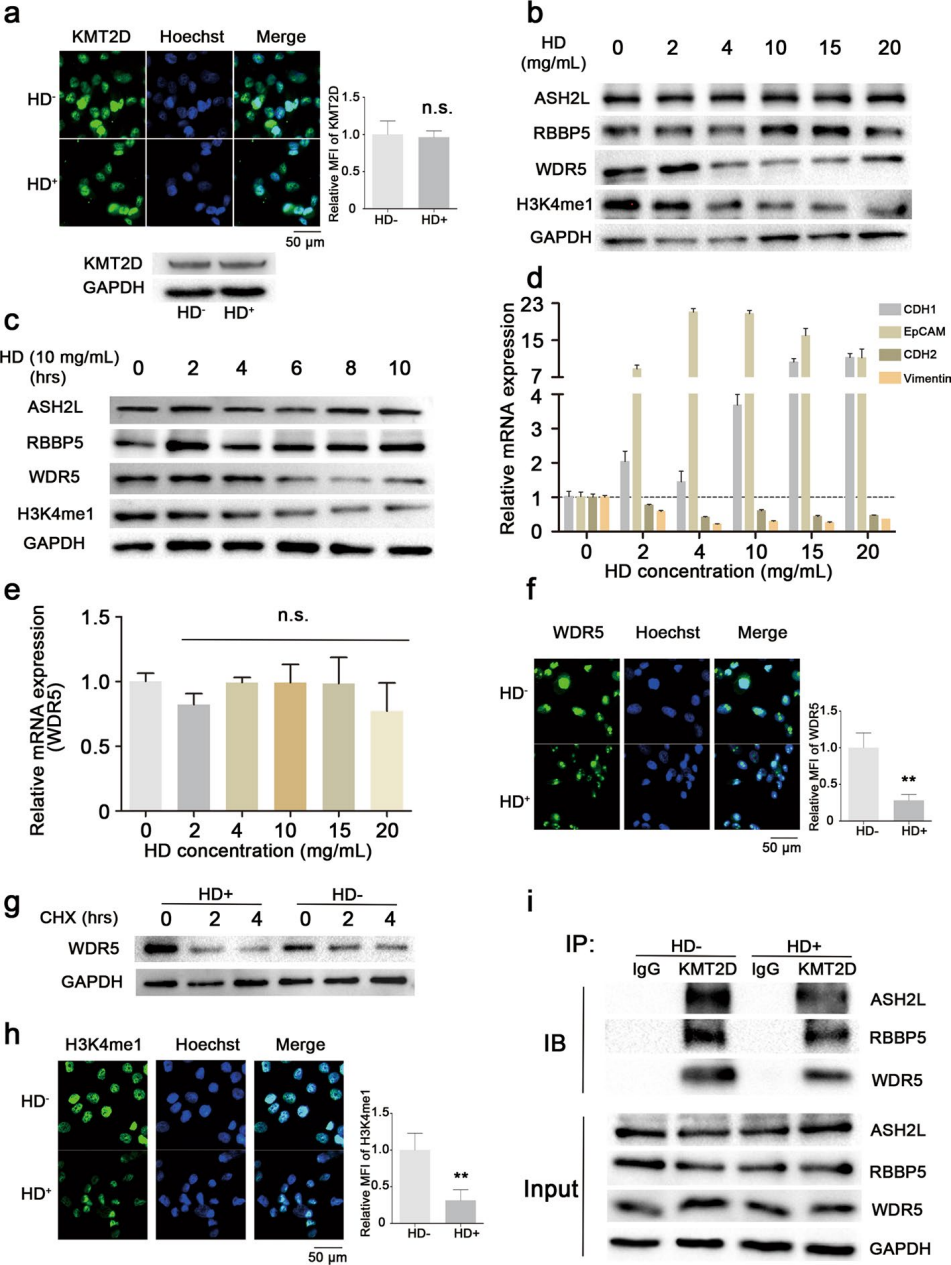
The LLPS microenvironment formed by the KMT2D protein influences its functions and modulates the stability of WDR5. **a** Representative images of cell immunofluorescence detecting the KMT2D expression in wild-type PANC-1 cells following treatment with 1,6-hexanediol (HD). KMT2D protein expression was markedly reduced and scattered after HD treatment. KMT2D, 593 KDa; GAPDH, 37 KDa. **b**, **c** Western blotting analyses of the KMT2D-enzyme complex in PANC-1 cells treated with HD. The expression of WDR5 decreased following HD treatment in a concentration- and time-dependent manner, while the expressions of ASH2L and RBBP5 were not affected. ASH2L, 69 KDa; RBBP5, 59 KDa; WDR5, 36 KDa; H3K4me1, 17 KDa; GAPDH, 37 KDa. **d** RT-PCR assays of metastasis-associated markers in PANC-1 cells treated with HD. The transcription levels of CDH1 and EpCAM significantly increased, while those of the CDH2 and vimentin decreased (error bars denote standard deviation). **e** RT-PCR assays of WDR5 in PANC-1 cells treated with HD. After HD treatment, the gene expression of WDR5 did not change (error bars denote standard deviation). **f** Representative images of cell immunofluorescence determining the WDR5 expression in PANC-1 cells treated with HD. The protein expression of WDR5 significantly decreased. **g** Western blotting analyses of the WDR5 expression after treatment with HD and CHX. Following HD treatment for 6 h at a concentration of 10 mg/mL, the stability of the WDR5 protein was sensitive to CHX. **h** Representative images of cell immunofluorescence determining the H3K4me1 expression in PANC-1 cells treated with HD. The level of H3K4me1 significantly decreased in cells treated with HD. **i** Co-immunoprecipitation analyses of the interactions between KMT2D and other components in PANC-1 cells treated with HD for 6 h at a concentration of 10 mg/mL. The interactions between KMT2D and WDR5, ASH2L and RBBP5 were markedly weaker following HD treatment. All values are represented as means ± SD. Data are representative of three independent experiments

Our results showed that the KMT2D expression in wild-type PANC-1 cells treated with HD was consistent with that in KMT2D LCD knockout PANC-1 cells (Fig. 6a and Additional file 1: Fig S6). The expression of WDR5 was shown to decrease in a concentration-dependent and time-dependent manner post HD treatment, while the expressions of other proteins (ASH2L and RBBP5) were not affected (Fig. 6b, c). Moreover, we also demonstrated that the stability of WDR5 protein decreased in siASH2L cells where the KMT2D-enzyme complex was impaired, indicating that the decrease in stability of WDR5 protein was due to impairing the formation of KMT2D-enzyme complex rather than direct inhibition of the protein by HD (Additional file 1: Fig. S7).

Interestingly, some phenomena associated with cell activities were in line with the results obtained from KMT2D LCD-deficient PANC-1 cells. For example, the transcription levels of epithelial markers (CDH1 and EpCAM) increased significantly, while those of the mesenchymal markers (CDH2 and vimentin) decreased after HD treatment at various concentrations, indicating that the LLPS microenvironment formed by the LCD structures of KMT2D could effectively alter the EMT activity of tumor cells (Fig. 6d).

RT-PCR results showed that the WDR5 RNA expression level remained unchanged in PANC-1 cells after HD treatment, suggesting that WDR5 transcription was not affected (Fig. 6e). By contrast, immunofluorescence images indicate that the expression of WDR5 protein significantly decreased compared to the control group (Fig. 6f). CHX treatment at a concentration of 50 µg/mL showed that the half-life of WDR5 protein in the cells treated with HD was significantly lower than for the control group (Fig. 6g). In addition, western blotting and immunofluorescence assays found that the level of H3K4me1 significantly decreased in wild-type PANC-1 cells treated with HD, indicating that KMT2D function was severely impaired (Fig. 6b, h). Protein–protein interaction results uncovered that the interactions between KMT2D and WDR5, ASH2L and RBBP5 were markedly weaker after HD treatment, which was consistent with the results obtained from KMT2D LCD knockout cells (Fig. 6i). In summary, our results demonstrate that HD treatment significantly impairs the function of the LCD domains of KMT2D by preventing the formation of the the LLPS microenvironment. This, in turn, significantly affects the stability of the WDR5 protein and further impacts the enzymatic complex formation and the monomethylation of H3K4.

**Fig. 7:**
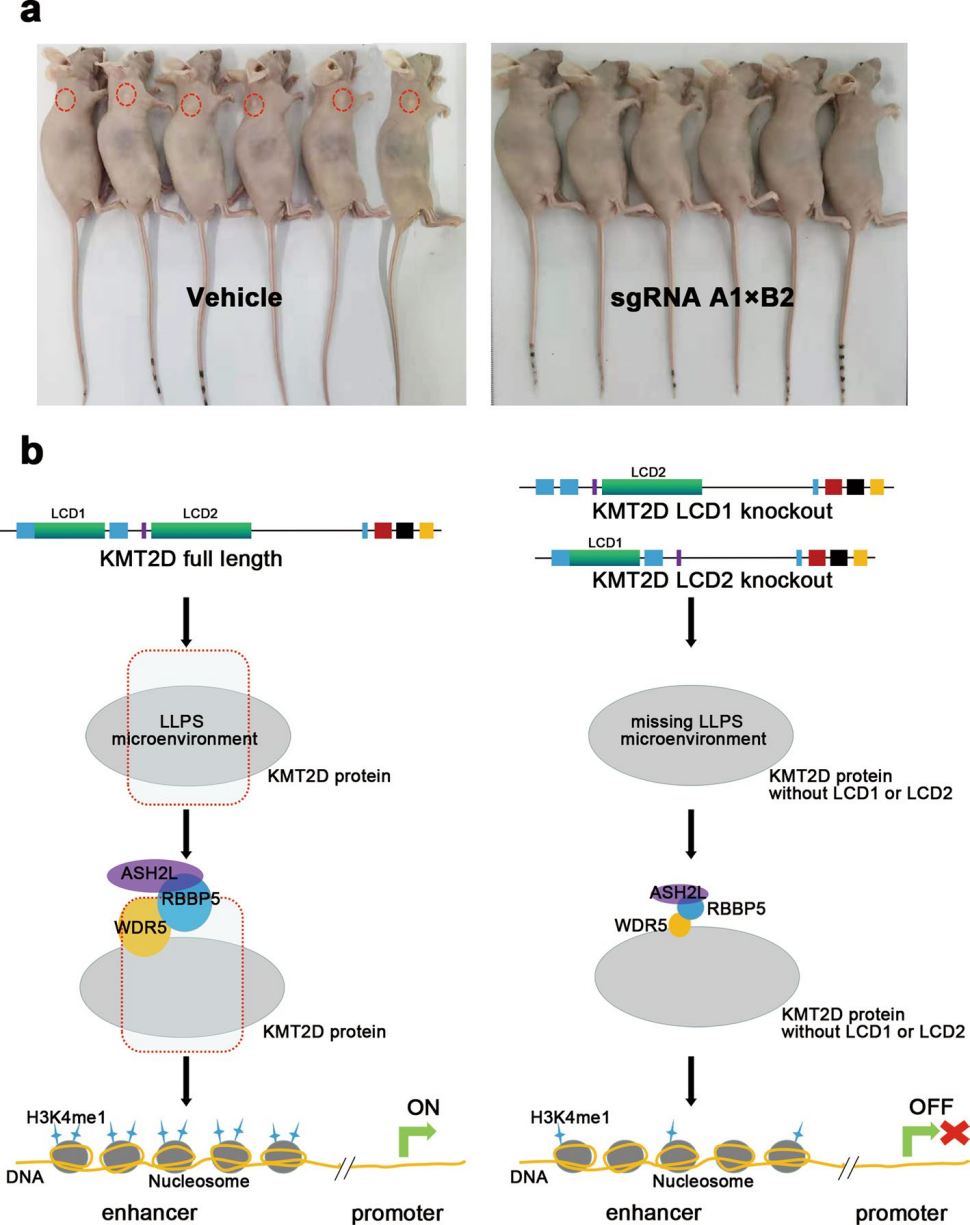
**a** Nude mice bearing established PANC1 xenograft tumors, including the vehicle group and sgRNA A1 × B2 group. After 2 weeks, tumor formation was monitored for a further 3 weeks. Interestingly, we could not detect tumor formation for PANC1 cells modified with LCD1 knockout, while the vehicle group could form tumors (red circle). **b** The proposed mechanistic model of for KMT2D LCDs in gene transcription. The knockout of KMT2D LCDs results in a loss of ability to form a stable LLPS microenvironment. The stability of WDR5 protein significantly decreased, and the protein–protein interactions between the components involved in the KMT2D–enzyme complex were attenuated. Some transcription factors, such as LIFR and KLF4, were markedly downregulated, attributed to the decrease in the H3K4me1 level. The left panel indicates the mechanism for full-length KMT2D, while the right panel indicates the mechanism for knocked out KMT2D LCDs

Next, we investigated the effect of KMT2D LCDs depletion on tumor formation using our mouse tumor model. The results demonstrate that the growth of xenograft tumors derived from KMT2D LCD-depleted cells (sgRNA A1×B2) was significantly reduced compared with the control cells (Fig. 7a). This shows that the LLPS microenvironment formed by the LCD domains of KMT2D is crucial to the enzymatic activity of KMT2D and further affects the occurrence and development of tumors.

## Discussion

With advances in the understanding of phase separation within the nucleus, research focus has turned to the role of the LLPS microenvironment in promoting the assembly of various transcription complexes, i.e., its role in gene expression [33–35]. The assembly of nuclear condensates has recently been recognized as a novel mechanism for driving gene expression, including transcription initiation condensates and RNA splicing condensates that respectively mediating pre-mRNA and mature RNA formation [36, 37].

H3K4me1 is a critical modulator of gene activation specifically catalyzed by KMT2D under physiological conditions. Coupled with the enzymatic functions of H3K27 demethylase and histone acetyltransferase p300 (H3K27Ac) at enhancers, the conversion of inactive enhancers to an active state is further facilitated [3]. Generally, for KMT2D to exert a histone monomethylation activity on unmethylated H3K4, there is a need to form an enzymatic complex with other components, including WDR5, ASH2L and RBBP5 [19, 38, 39]. Based on our analyses, KMT2D is the only protein involved in this complex with two distinct LCD structures that are able to form the LLPS microenvironment. The other components of the KMT2D–enzyme complex do not have this ability and this is attributed to their lack of any obvious LCD regions (Additional file 1: Fig. S8). Therefore, studying the function of KMT2D LCDs provides a basis for investigating the effects of LLPS on gene transcription. To the best of our knowledge, we are first to report that the LCD structures of the KMT2D protein could significantly affect histone modification, characterized by the increase in the H3K4me1 level and the facilitation of gene transcription.

Subsequently, we systematically explored the mechanism whereby KMT2D LCDs regulate the expression of H3K4me1 and transcription factors related to tumor proliferation and metastasis. As depicted in Fig. 7b, the knockout of KMT2D LCDs removes the ability to form a stable LLPS microenvironment. Without the LLPS microenvironment, the stability of KMT2D and WDR5 protein significantly decreased, and the protein–protein interactions between the components involved in KMT2D–enzyme complex were attenuated, impairing its formation. With the decrease in H3K4me1 level at enhancers, transcription factors such as LIFR and KLF4 were markedly downregulated, effectively inhibiting tumor progression. Notably, our demonstration of the stabilizing effect of KMT2D LCD function through the formation of the LLPS microenvironment provides a mechanistic basis for better understanding the monomethyltransferase activity of KMT2D protein.

Our study offers new insight into the functional mechanism of the KMT2D protein in tumor survival. There are two LCD structures in KMT2D protein that allow it to easily form the LLPS microenvironment around gene enhancers. By identifying some downstream targets regulated by KMT2D protein, combined with functional studies including the H3K4me1 level, we robustly defined that KMT2D functions are strictly dependent on the integrity of its LCDs. This study revealed a mechanism for KMT2D LCD structures affecting H3K4me1 modification, and thus affecting the stability of WDR5 protein and the formation of the KMT2D–enzyme complex. We also demonstrated that KMT2D LCDs further significantly affect the expression of some tumor-related transcription factors, including LIFR and KLF4, which are respectively associated with tumor proliferation and metastasis signaling pathways, thus providing a rationale for studying the function of the LLPS microenvironment derived from KMT2D LCDs in tumorigenesis. Our study further corroborates the importance of the LLPS microenvironment in gene transcription, and more importantly, provides a potential novel strategy for epigenetic therapy in treating pancreatic cancer.

## Conclusions

Based on these findings, the two low-complexity domain structures in KMT2D protein can form a stable LLPS microenvironment around the gene enhancers. Furthermore KMT2D can catalyze H3K4 monomethylation by stabilizing the WDR5 protein and enhancing the interactions between components within the KMT2D–enzyme complex in an LLPS-dependent manner, thus promoting cancer progression. We have here shown that the LLPS microenvironment has a key regulating effect on gene transcription and could be used as a target in cancer treatment.

## Supplementary Information

**Additional file 1: Table S1.** Primers used in this study. **Fig. S1.** The Kaplan-Meier curve of recurrence-free survival analysis to assess the prognostic value of KMT2D expression in the GEO database. **Fig. S2.** Identification of genomic DNA isolated from HEK293T and PANC-1 cells transfected with sgRNA plasmids. **Fig. S3.** Representative images of western blots in HEK293T and PANC-1 cells. **Fig. S4.** Detection of H3K4me1 expression in HEK293T cells. **Fig. S5.** Expression levels of WDR5 in HEK293T cells. **Fig. S6.** Detection of the LLPS microenvironment in KMT2D protein. **Fig. S7.** The decreased stability of WDR5 protein was due to the impaired formation of the KMT2D–enzyme complex rather than direct inhibition of the protein by 1,6-hexanediol (HD). **Fig. S8.** Prediction of the low complexity domains of proteins involved in KMT2D–enzyme complex using the PONDR database.

## Data Availability

All data generated or analyzed during this study are included in this published article (and its Additional file 1).

## Abbreviations

H3K4: Histone 3 lysine 4
H3K4me1: Monomethylated H3K4
H3K4me2: Di-methylated H3K4
H3K4me3: Tri-methylated H3K4
KMT2D: Histone lysine methyltransferase 2D
LIFR: Leukemia inhibitory factor receptor
KLF4: Kruppel-like factor-4
EMT: Epithelial-to-mesenchymal transition
PHD: Plant homeotic domain
FYRN: Phenylalanine and tyrosine-rich N-terminal
FYRC: Phenylalanine and tyrosine-rich C-terminal
HMG-I: High-mobility group I
LLPS: Liquid–liquid phase separation
IDR: Intrinsically disordered region
LCD: Low-complexity domain
Trr: Trithorax-related
KMT2: Histone–lysine N-methytransferase 2
WDR5: WD repeat protein 5
RBBP5: Retinoblastoma-binding protein 5
ASH2L: Absent small homeotic 2 like
DPY30: Dumpy-30
NCOA6: Nuclear receptor coactivator 6
CHX: Cycloheximide
HD: 1,6-Hexanediol
