# Supplementary material for: The low-complexity domains of the KMT2D protein regulate histone monomethylation transcription to facilitate pancreatic cancer progression
Source: Cell Mol Biol Lett. 2021 Nov 10;26:45. doi: 10.1186/s11658-021-00292-7 (PMC8579673; doi:10.1186/s11658-021-00292-7)
Supplement: Supplementary file 1 — Additional file 1: Table S1. Primers used in this study. Fig. S1. The Kaplan-Meier curve of recurrence-free survival analysis to assess the prognostic value of KMT2D expression in the GEO database. Fig. S2. Identification of genomic DNA isolated from HEK293T and PANC-1 cells transfected with sgRNA plasmids. Fig. S3. Representative images of western blots in HEK293T and PANC-1 cells. Fig. S4. Detection of H3K4me1 expression in HEK293T cells. Fig. S5. Expression levels of WDR5 in HEK293T cells. Fig. S6. Detection of the LLPS microenvironment in KMT2D protein. Fig. S7. The decreased stability of WDR5 protein was due to the impaired formation of the KMT2D–enzyme complex rather than direct inhibition of the protein by 1,6-hexanediol (HD). Fig. S8. Prediction of the low complexity domains of proteins involved in KMT2D–enzyme complex using the PONDR database. [file 11658_2021_292_MOESM1_ESM.docx]

**Additional file 1**

**The low-complexity domains of the KMT2D protein regulate histone monomethylation transcription to facilitate pancreatic cancer progression**

Weihan Li^1, #^, Lei Wu^1, #^, Hui Jia^1, 2, #^, Zenghua Lin^3^, Renhao Zhong^1, 2^, Yukun Li^1, 2^, Chenwei Jiang^1, 2^, Shifan Liu^1, 2^, Xiaorong Zhou^1,^ * and Erhao Zhang^1, 2,^ *

^1^Department of Immunology, School of Medicine, Nantong University, Nantong 226001, P.R. China

^2^Laboratory of Medical Science, School of Medicine, Nantong University, Nantong 226001, P.R. China

^3^Department of Hematology, Affiliated Hospital of Nantong University, Nantong University, Nantong 226001, P.R. China

^#^These authors contributed equally to this work.

**Correspondence:** Erhao Zhang, Email: zhangerhao@ntu.edu.cn; Xiaorong Zhou, Email: zhouxiaorong@ntu.edu.cn

**Table S1.** Primers in this research.

| Primer name | Sequence (5’→3’) | Application |
| --- | --- | --- |
| H-KMT2D-A1-F | ccggggacaatagggcagaatca | sgRNA |
| H-KMT2D-A1-R | aactgattctgccctattgtccc | sgRNA |
| H-KMT2D-A2-F | ccgggagtgggcaaaacaggcat | sgRNA |
| H-KMT2D-A2-R | aacatgcctgttttgcccactcc | sgRNA |
| H-KMT2D-B1-F | ccggtgaggggctatctagctgc | sgRNA |
| H-KMT2D-B1-R | aacgcagctagatagcccctcac | sgRNA |
| H-KMT2D-B2-F | ccgcaggactgtacctctgacag | sgRNA |
| H-KMT2D-B2-R | aacctgtcagaggtacagtcctg | sgRNA |
| H-KMT2D-1-F | gctcactcttggaagtgcaag | Genotyping PCR |
| H-KMT2D-1-R | caattctattactgagtaacc | Genotyping PCR |
| H-KMT2D-C1-F | ccggggtgtagcagtcctctagt | sgRNA |
| H-KMT2D-C1-R | aacactagaggactgctacaccc | sgRNA |
| H-KMT2D-C2-F | ccggtggcctctcttgagggtgg | sgRNA |
| H-KMT2D-C2-R | aacccaccctcaagagaggccac | sgRNA |
| H-KMT2D-D1-F | ccggtaagtggtcaggtgggagt | sgRNA |
| H-KMT2D-D1-R | aacactcccacctgaccacttac | sgRNA |
| H-KMT2D-D2-F | ccggaacttgtgtcttatgccac | sgRNA |
| H-KMT2D-D2-R | aacgtggcataagacacaagttc | sgRNA |
| H-KMT2D-2-F | cgcatcaacaaggtgcagaag | Genotyping PCR |
| H-KMT2D-2-R | gggatcaccagcactccgctc | Genotyping PCR |
| H-PDLIM7-F | acagccgctccgaccgctggt | Genotyping PCR |
| H-PDLIM7-R | ggcaaggatgcggaaggaacg | Genotyping PCR |
| H-LCD1-F | ctctatgcaacccaaggaac | RT-PCR |
| H-LCD1-R | aactggggctcaagttgg | RT-PCR |
| H-LCD2-F | ttcttcccagatacagacct | RT-PCR |
| H-LCD2-R | ctttcaaagccaccatcttg | RT-PCR |
| H-SET-F | gagcctaaaatcctcacaca | RT-PCR |
| H-SET-R | tgtgaaggtgctctgatatg | RT-PCR |
| H-CDH1-F | ggagaagaggaccaggactt | RT-PCR |
| H-CDH1-R | agtatcagccgctttcagat | RT-PCR |
| H-EpCAM-F | tggggaacaactggatctgg | RT-PCR |
| H-EpCAM-R | ccagcacaacaattccagca | RT-PCR |
| H-CDH2-F | gagacattggggacttcatt | RT-PCR |
| H-CDH2-R | caaacactaacagggagtca | RT-PCR |
| H-Vimentin-F | tgtttccaagcctgacctca | RT-PCR |
| H-Vimentin-R | tgtctccggtactcagtgga | RT-PCR |
| H-KLF4-F | acacaaagagttcccatctc | RT-PCR |
| H-KLF4-R | tagtgcctggtcagttcat | RT-PCR |
| H-LIFR-F | tattgccattctcatcccag | RT-PCR |
| H-LIFR-R | ctggatttggaatatcagggt | RT-PCR |
| H-WDR5-F | aaactacaaggccacacaga | RT-PCR |
| H-WDR5-R | tagcagtcactcttccacag | RT-PCR |
| H-GAPDH-F | gaaggactcatgaccacag | RT-PCR |
| H-GAPDH-R | gcagggatgatgttctgg | RT-PCR |

**
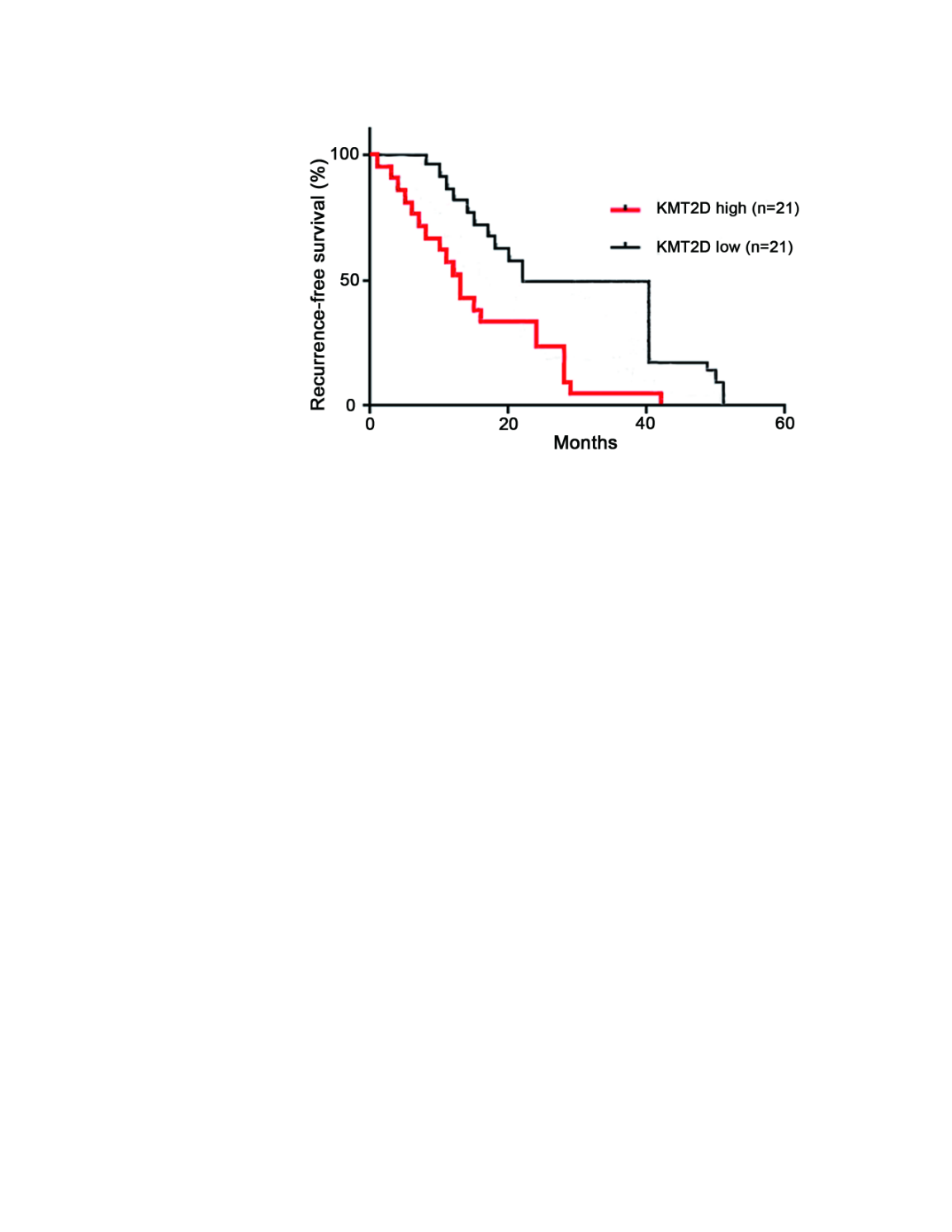
**

**Figure S1.** The Kaplan-Meier curve of recurrence-free survival analysis to assess prognostic value of KMT2D expression in the GEO database. Lower expression of KMT2D (black) correlates with improved patient survival compared with higher expression (red) in pancreatic ductal adenocarcinoma.

**
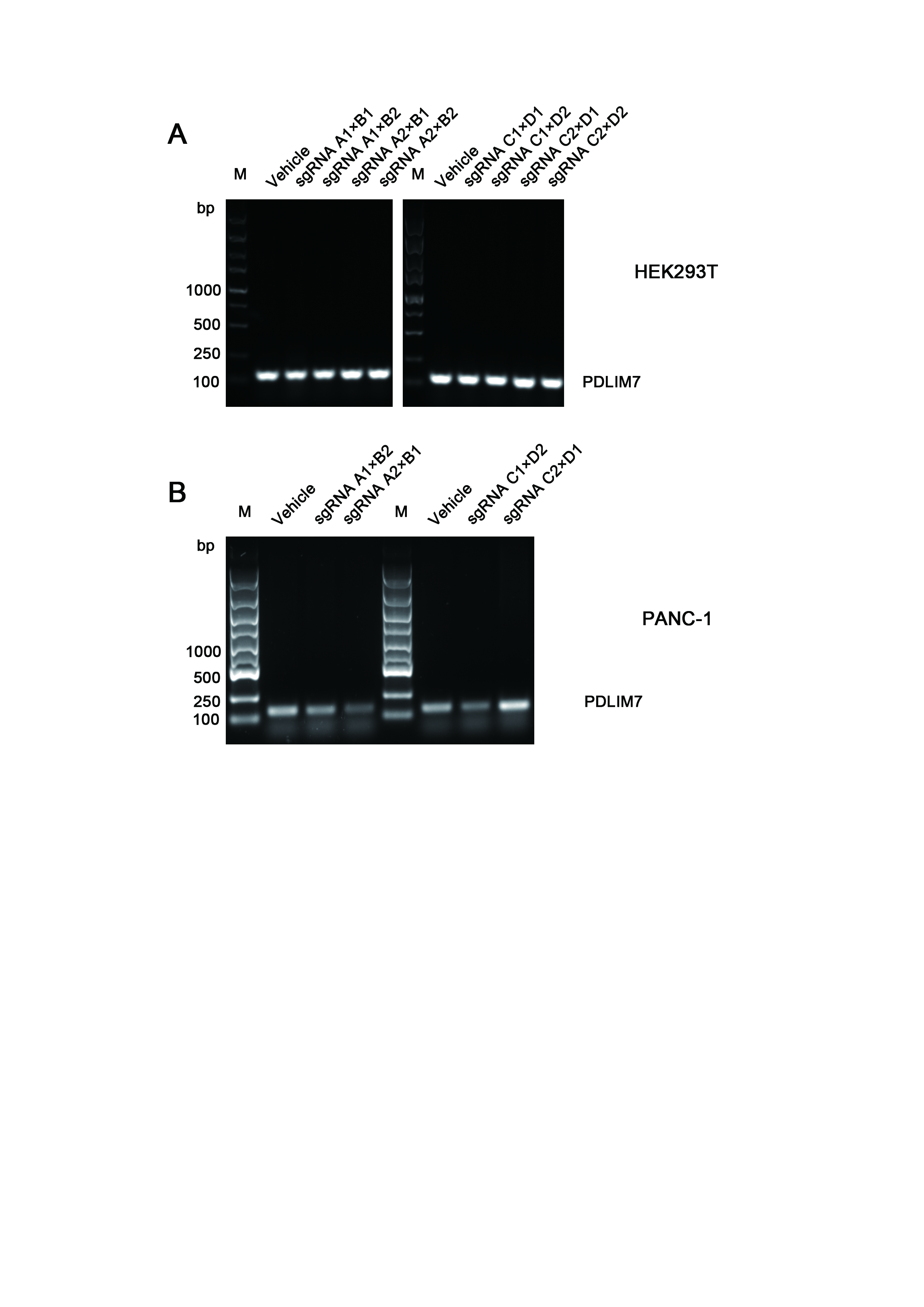
**

**Figure S2.** Identification of genomic DNA isolated from HEK293T and PANC-1 cells transfected with sgRNA plasmids. The cell DNA was extracted, and then the control gene, PDLIM7, was amplified by PCR. **a** HEK293T cell; **b** PANC-1 cell.

**
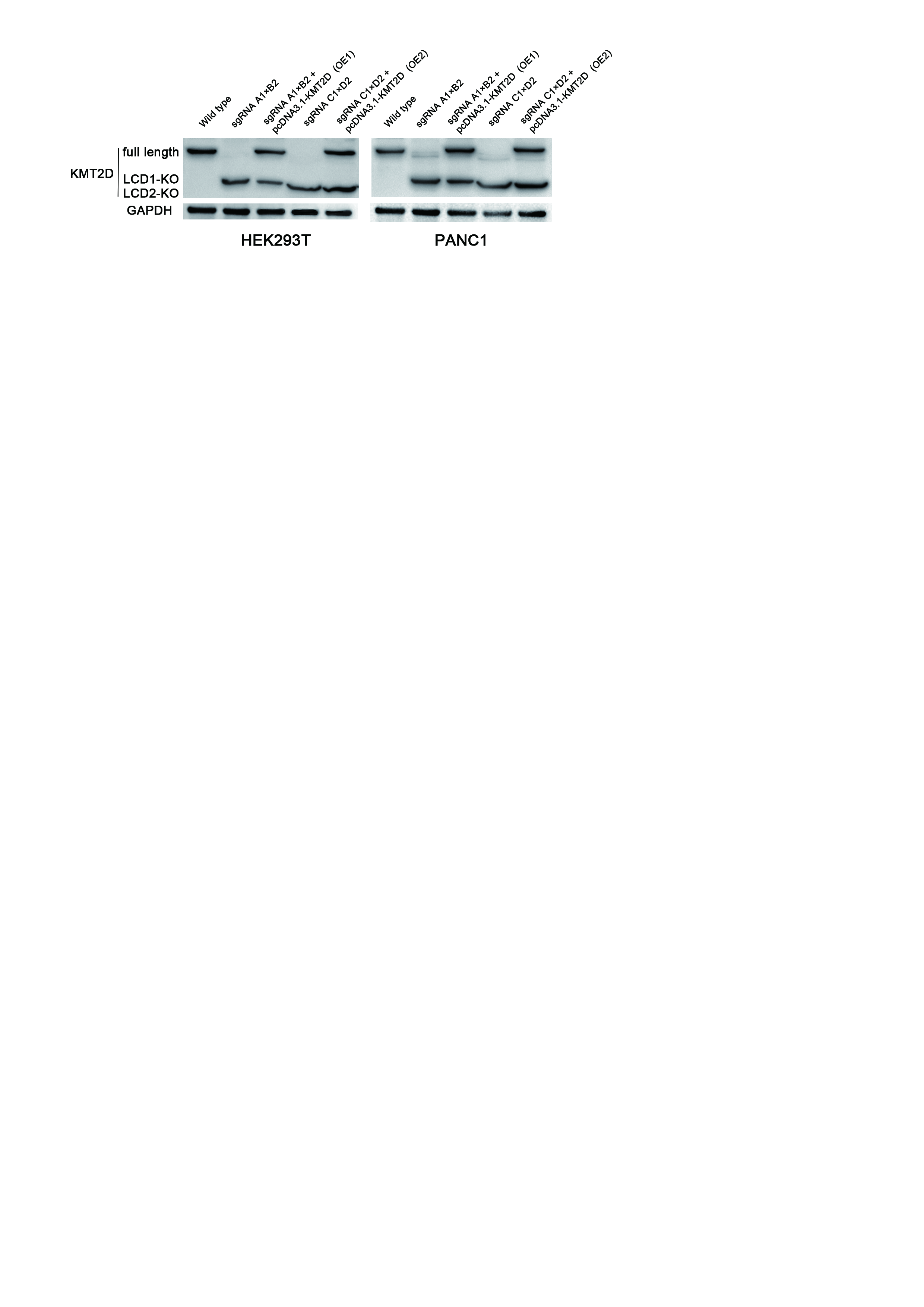
**

**Figure S3.** Representative images of western blot in HEK293T and PANC-1 cells. After LCDs knockout, KMT2D protein has no significant decrease, and KMT2D-LCD1 or KMT2D-LCD2 is deleted. Importantly, in OE1 cells and OE2 cells, the KMT2D-LCDs deleted cells could successfully re-express the KMT2D protein full length. KMT2D, 593 KDa; LCD1-deleted KMT2D (LCD1-KO), 483 KDa; LCD2-deleted KMT2D (LCD2-KO), 447 KDa.

**
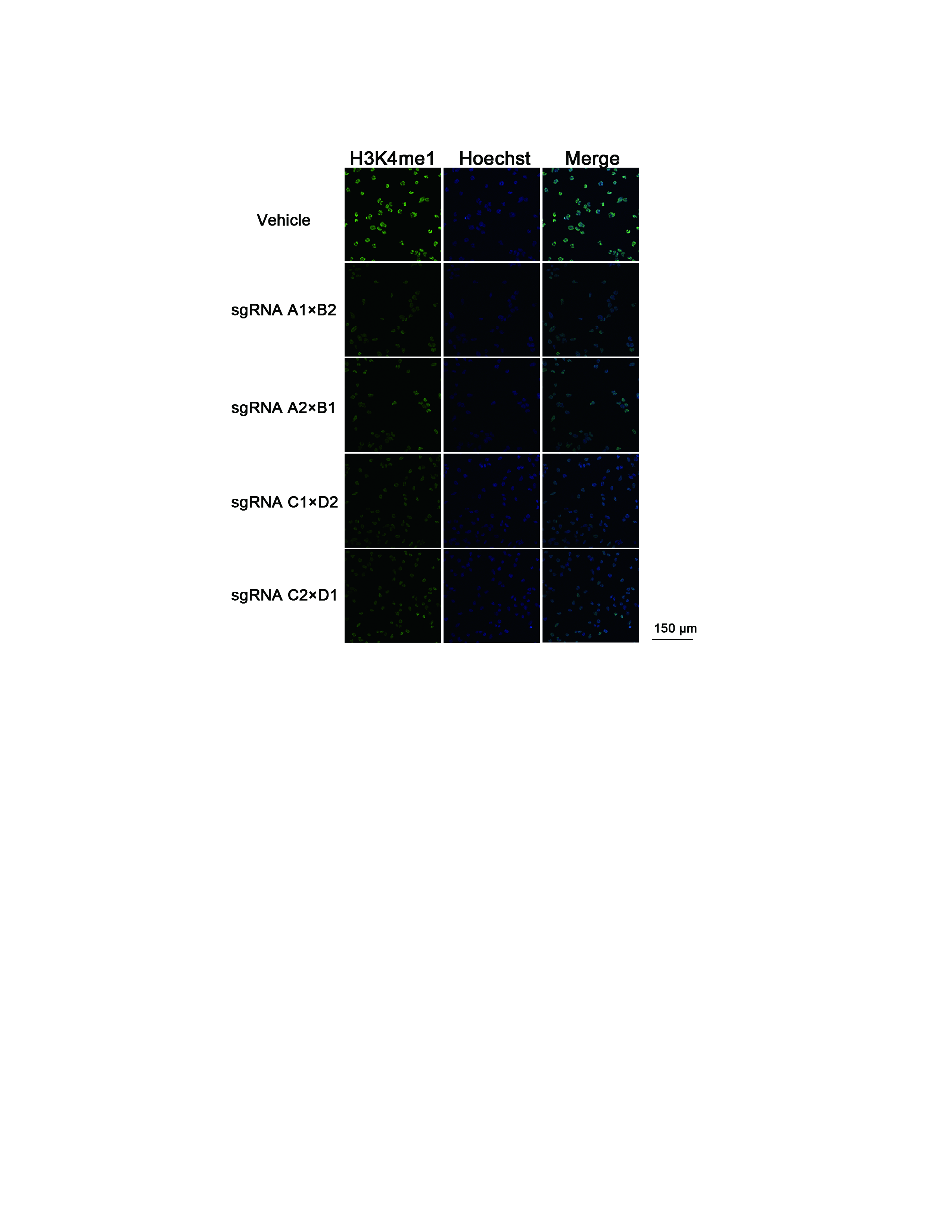
**

**Figure S4.** Detection of H3K4me1 expression in HEK293T cells. Representative images of cell immunofluorescence detecting the H3K4me1 expression in HEK293T cells. After LCDs knockout, the H3K4me1 level was remarkably reduced in the nucleus.

**
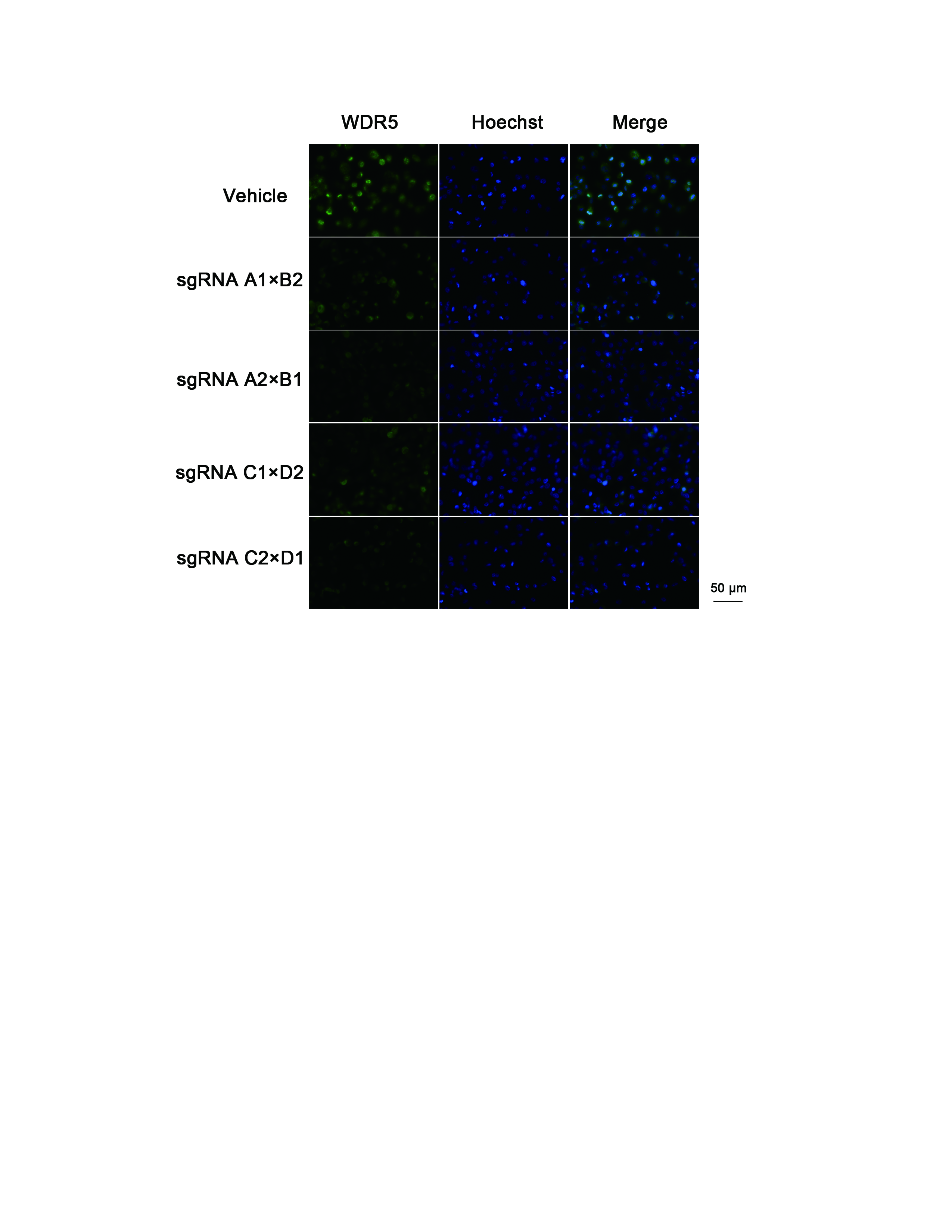
**

**Figure S5.** Expression levels of WDR5 in HEK293T cells. Representative images of cell immunofluorescence detecting the WDR5 expression in modified-HEK293T cells. The WDR5 level was remarkably reduced in the LCDs-deleted cells.

**
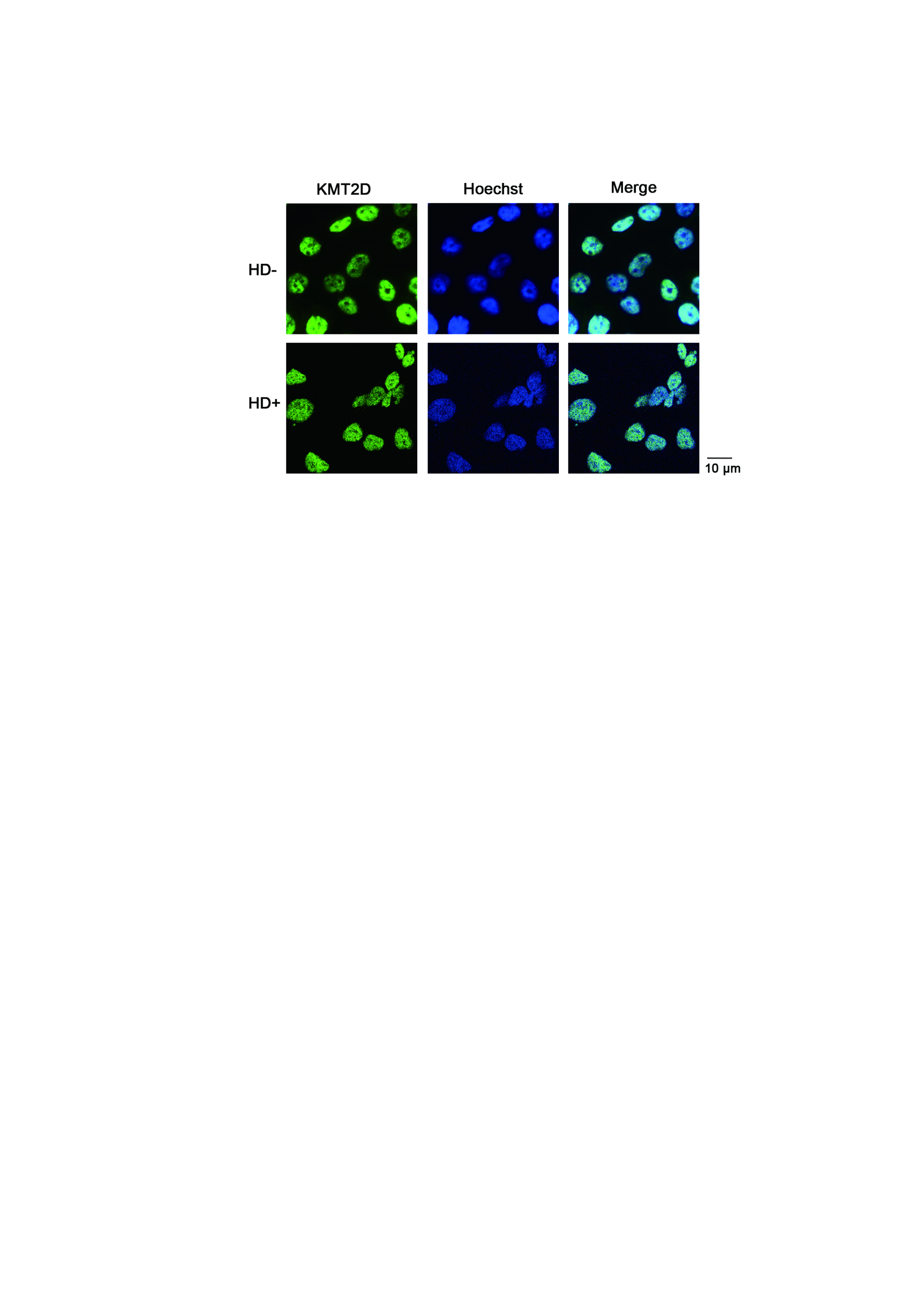
**

**Figure S6.** Detection of the LLPS microenvironment in KMT2D protein. Representative images of cell immunofluorescence detecting the KMT2D protein in PANC-1 cells with the treatment of 1,6-hexanediol (HD), an inhibitor of LLPS formation. With the treatment of HD (lower panel), cell immunofluorescence indicated that KMT2D protein in the cells modified with LCDs knockout was remarkably scattered, while that of the control group (vehicle) was clustered in the nucleus.


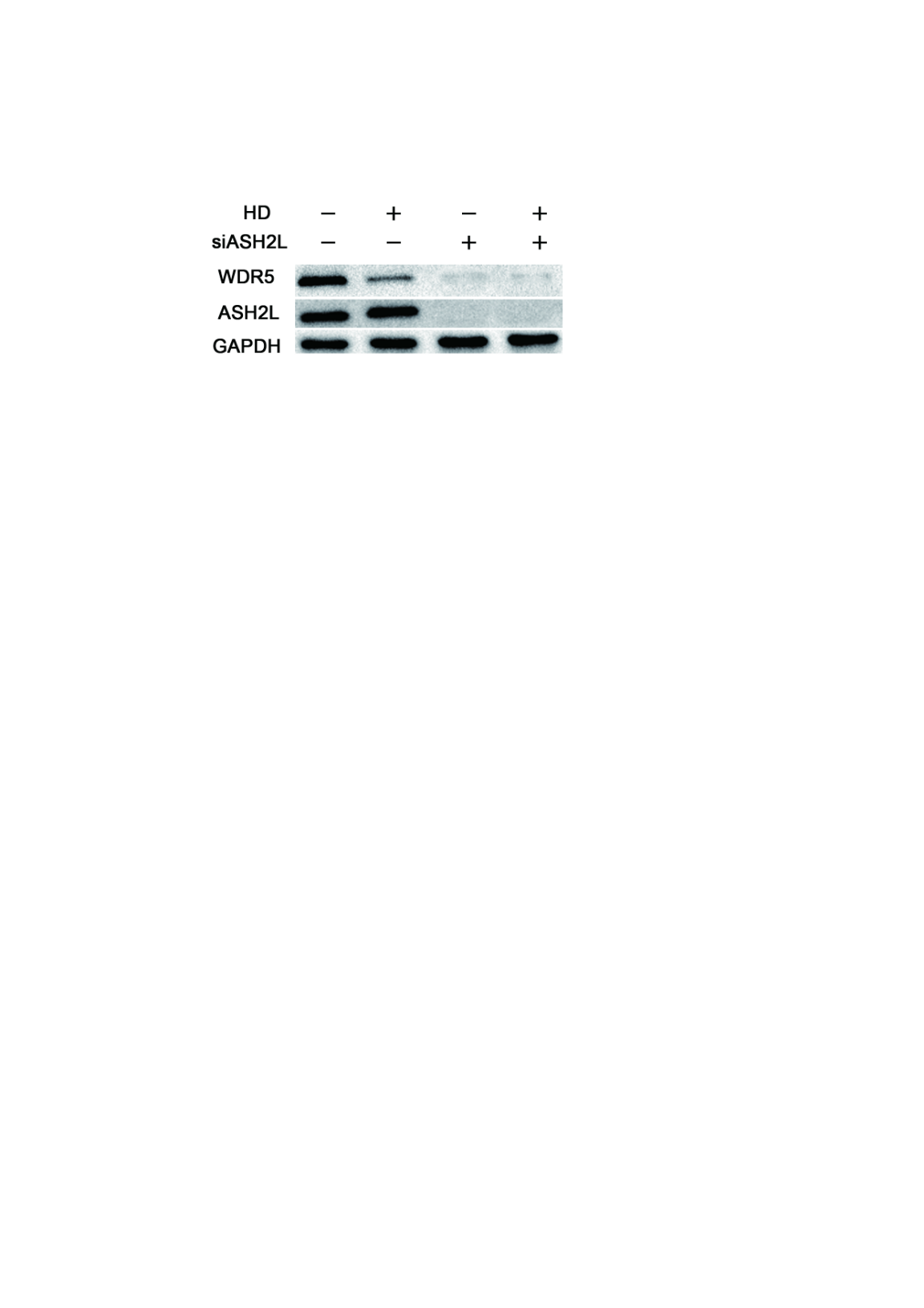


**Figure S7.** The decreased stability of WDR5 protein was due to the impaired formation of the KMT2D-enzyme complex rather than direct inhibition of the protein by 1,6-hexanediol (HD).


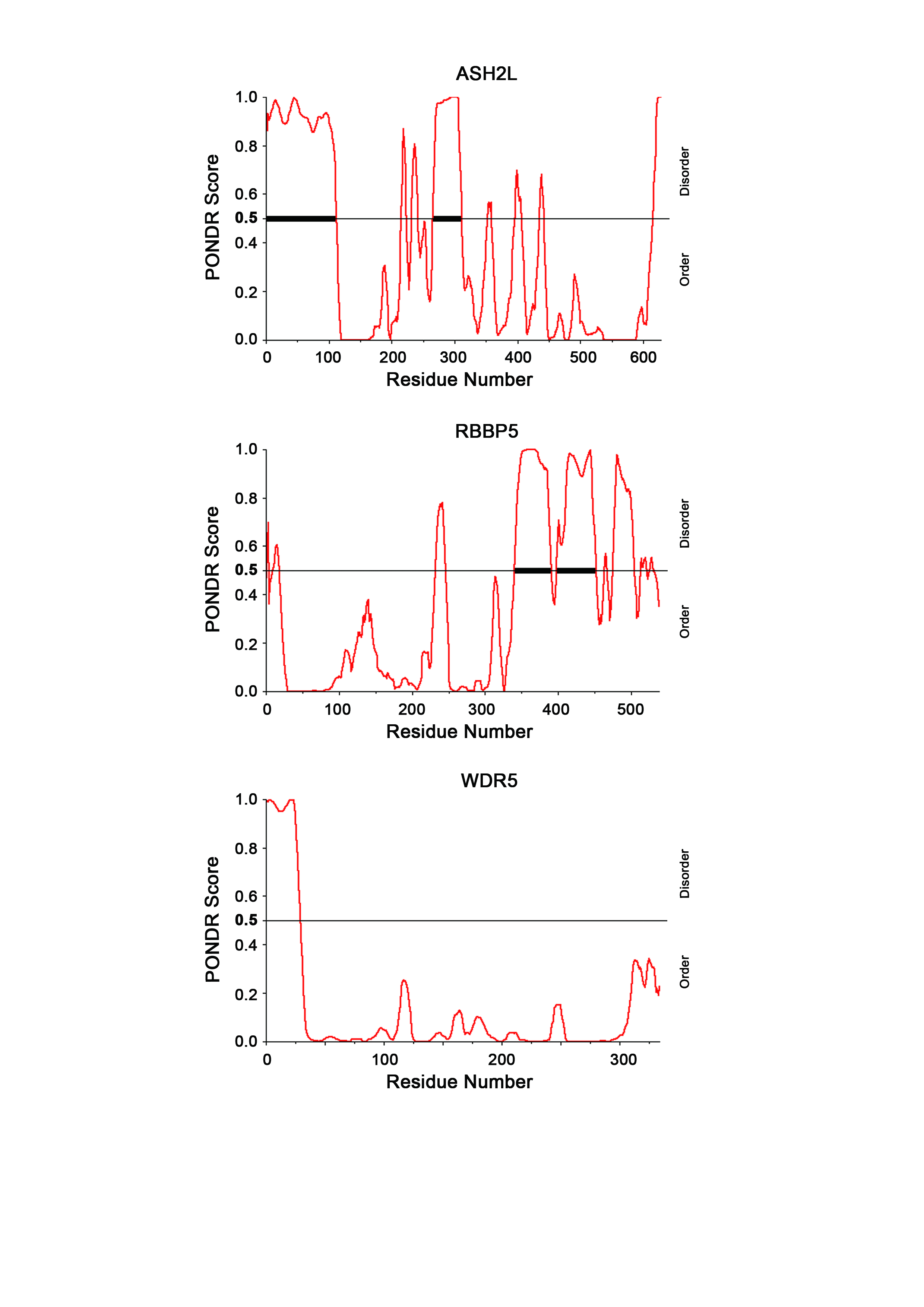


**Figure S8.** Prediction of low complexity domains of proteins involved in KMT2D-enzyme complex using the PONDR database. Upper panel: ASH2L protein; Middle panel: RBBP5 protein; Lower panel: WDR5 protein.
